# Identification of Dephospho-Coenzyme A (Dephospho-CoA) Kinase in Thermococcus kodakarensis and Elucidation of the Entire CoA Biosynthesis Pathway in Archaea

**DOI:** 10.1128/mBio.01146-19

**Published:** 2019-07-23

**Authors:** Takahiro Shimosaka, Kira S. Makarova, Eugene V. Koonin, Haruyuki Atomi

**Affiliations:** a Department of Synthetic Chemistry and Biological Chemistry, Graduate School of Engineering, Kyoto University, Kyoto, Japan; b Japan Society for the Promotion of Science, Tokyo, Japan; c National Center for Biotechnology Information, National Library of Medicine, National Institutes of Health, Bethesda, Maryland, USA; University of Vienna

**Keywords:** archaea, coenzyme A, dephospho-CoA kinase, hyperthermophiles, metabolism

## Abstract

Dephospho-coenzyme A (dephospho-CoA) kinase (DPCK) catalyzes the ATP-dependent phosphorylation of dephospho-CoA, the final step in coenzyme A (CoA) biosynthesis. DPCK has been identified and characterized in bacteria and eukaryotes but not in archaea. The hyperthermophilic archaeon Thermococcus kodakarensis encodes two homologs of bacterial DPCK and the DPCK domain of eukaryotic CoA synthase, TK1334 and TK2192. We purified the recombinant TK1334 and TK2192 proteins and found that they lacked DPCK activity. Bioinformatic analyses showed that, in several archaea, the uncharacterized gene from arCOG04076 protein is fused with the gene for phosphopantetheine adenylyltransferase (PPAT), which catalyzes the reaction upstream of the DPCK reaction in CoA biosynthesis. This observation suggested that members of arCOG04076, both fused to PPAT and standalone, could be the missing archaeal DPCKs. We purified the recombinant TK1697 protein, a standalone member of arCOG04076 from T. kodakarensis, and demonstrated its GTP-dependent DPCK activity. Disruption of the TK1697 resulted in CoA auxotrophy, indicating that TK1697 encodes a DPCK that contributes to CoA biosynthesis in T. kodakarensis. TK1697 homologs are widely distributed in archaea, suggesting that the arCOG04076 protein represents a novel family of DPCK that is not homologous to bacterial and eukaryotic DPCKs but is distantly related to bacterial and eukaryotic thiamine pyrophosphokinases. We also constructed and characterized gene disruption strains of TK0517 and TK2128, homologs of bifunctional phosphopantothenoylcysteine synthetase-phosphopantothenoylcysteine decarboxylase and PPAT, respectively. Both strains displayed CoA auxotrophy, indicating their contribution to CoA biosynthesis. Taken together with previous studies, the results experimentally validate the entire CoA biosynthesis pathway in T. kodakarensis.

## Abstract (precis)

CoA is utilized in a wide range of metabolic pathways, and its biosynthesis is essential for all life. Pathways for CoA biosynthesis in bacteria and eukaryotes have been established. In archaea, however, the enzyme that catalyzes the final step in CoA biosynthesis, dephospho-CoA kinase (DPCK), had not been identified. In the present study, bioinformatic analyses identified a candidate for the DPCK in archaea, which was biochemically and genetically confirmed in the hyperthermophilic archaeon Thermococcus kodakarensis. Genetic analyses on genes presumed to encode bifunctional phosphopantothenoylcysteine synthetase-phosphopantothenoylcysteine decarboxylase and phosphopantetheine adenylyltransferase confirmed their involvement in CoA biosynthesis. Taken together with previous studies, the results reveal the entire pathway for CoA biosynthesis in a single archaeon and provide insight into the different mechanisms of CoA biosynthesis and their distribution in nature.

## INTRODUCTION

**FIG 1:**
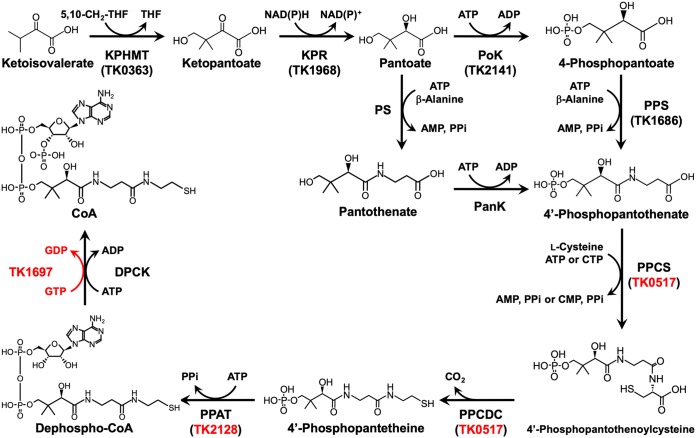
CoA biosynthesis pathways in the three domains of life. The conversion of pantoate to 4′-phosphopantothenate is catalyzed by PS and PanK in bacteria and eukaryotes. PoK and PPS replace the PS-PanK system in most archaea. Genes that encode CoA biosynthesis enzymes in T. kodakarensis are noted in parentheses. The reaction catalyzed by the novel DPCK encoded by the TK1697 gene is indicated in red, along with the genes (TK0517 and TK2128) evaluated in this study. THF, tetrahydrofolate. Other abbreviations are defined in the text.

Coenzyme A (CoA) is an essential cofactor found in all three domains of life. CoA forms high-energy thioester bonds with various carbonyl compounds and is involved in a wide range of metabolic pathways that include the tricarboxylic acid cycle and β-oxidation, as well as fatty acid and isoprenoid biosynthesis (1–3). In bacteria and eukaryotes, CoA is synthesized from pantothenate via 5 consecutive reactions that are catalyzed by pantothenate kinase (PanK), phosphopantothenoylcysteine synthetase (PPCS), phosphopantothenoylcysteine decarboxylase (PPCDC), phosphopantetheine adenylyltransferase (PPAT), and dephospho-CoA kinase (DPCK) (Fig. 1). In plants and the majority of bacteria, pantothenate can be synthesized from ketoisovalerate and β-alanine via three additional reactions catalyzed by ketopantoate hydroxymethyltransferase (KPHMT), ketopantoate reductase (KPR), and pantothenate synthetase (PS). Animals and a minority of bacteria do not have the ability to convert ketoisovalerate to pantothenate and must rely on exogenous pantothenate for CoA synthesis.

Until recently, the pathway for CoA biosynthesis in archaea remained largely uncharacterized, but several of the constituent enzymes have been identified in the past decade. The first four enzymes of the CoA biosynthesis pathway converting ketoisovalerate to 4′-phosphopantothenate and the protein necessary for β-alanine synthesis have been identified and characterized in Thermococcus kodakarensis (4–8). T. kodakarensis is a hyperthermophilic archaeon in the phylum *Euryarchaeota* (9, 10). Its genome sequence has been determined (11), and a versatile genetic system has been developed (12–16). Among the four enzymes, pantoate kinase (PoK) and phosphopantothenate synthetase (PPS) are unique to the archaea and replace PS and PanK in bacteria and eukaryotes in the conversion of pantoate to 4′-phosphopantothenate (8) (Fig. 1). The presence of PoK and PPS has also been demonstrated in Methanospirillum hungatei (17). PoK and PPS homologs are encoded in the majority of archaeal genomes, with exceptions limited to members of the *Nanoarchaeota*, *Nanohaloarchaeota*, *Korarchaeota*, *Bathyarchaeota*, and *Thermoplasmatales*. A PanK has been identified in Picrophilus torridus, raising the possibility that members of the *Thermoplasmatales* utilize a pathway similar to that in bacteria and eukaryotes (18). As for the enzymes that act downstream of 4′-phosphopantothenate, a bifunctional PPCS-PPCDC and PPAT have been identified and characterized in the hyperthermophilic methanogen Methanocaldococcus jannaschii (19) and in the hyperthermophilic euryarchaeon Pyrococcus abyssi (20), respectively. T. kodakarensis encodes homologs of this PPCS-PPCDC and PPAT that are encoded by TK0517 and TK2128, respectively. In terms of regulation of CoA biosynthesis, PanK acts as the target of feedback inhibition in the pathways in bacteria and eukaryotes. As described above, most pathways in archaea do not utilize PanK, and instead, in T. kodakarensis, KPR is inhibited in the presence of CoA (5). Although progress has been made in understanding the mechanisms of CoA biosynthesis in archaea, DPCK that catalyzes the final reaction, the phosphorylation of dephospho-CoA, so far has not been identified in any of the archaea.

Here, we describe the identification and experimental characterization of a novel DPCK, encoded by the TK1697 gene of T. kodakarensis. This enzyme is not homologous to the classical DPCK from bacteria and eukaryotes but is distantly related to bacterial and eukaryotic thiamine pyrophosphokinases. Orthologs of TK1697 are widely distributed in archaea, suggesting that this form of DPCK is responsible for the last step of CoA biosynthesis in most of the archaea. In addition, we genetically confirmed the involvement of TK0517, a homolog of PPCS-PPCDC, and TK2128, a homolog of PPAT, in CoA biosynthesis in T. kodakarensis. Together with the results of previous studies, this work completes the elucidation of the entire pathway for CoA biosynthesis in T. kodakarensis and, by inference, in other archaea.

## RESULTS

### Expression, purification, and examination of the recombinant TK1334 and TK2192 proteins.

The T. kodakarensis genome harbors two genes, TK1334 and TK2192, which are annotated as DPCK. Indeed, the TK1334 and TK2192 proteins show highly statistically significant albeit relatively low (e.g., 14% and 16% identity with the Escherichia coli DPCK, respectively) similarity to bacterial and eukaryotic DPCK sequences. In order to examine whether either of these genes encoded proteins with DPCK activity, the genes were individually expressed in E. coli, and the recombinant proteins were purified. The samples were subjected to SDS-PAGE, and single bands corresponding to the calculated molecular masses of TK1334 (20,844 Da) and TK2192 (22,078 Da) were observed in each lane (see Fig. S1A and B in the supplemental material), indicating that each protein was purified to apparent homogeneity. Using the purified recombinant proteins, DPCK activity was assayed. However, no CoA generation was observed with these recombinant proteins when incubated with dephospho-CoA and ATP. These results indicate that TK1334 and TK2192, although homologous to bacterial and eukaryotic DPCK, are involved in different pathways that remain to be identified. Given the essentiality of the DPCK reaction, this finding implies that the true DPCK in T. kodakarensis is encoded by an unidentified gene.

10.1128/mBio.01146-19.1 FIG S1 SDS-PAGE analysis of the purified TK1334 (A), TK2192 (B), and TK1697 (C) proteins. The amount of proteins applied in panels A and B was 1 μg, and that for panel C was 0.5 μg. Gels were stained with Coomassie brilliant blue. Download FIG S1, TIF file, 0.7 MB. Copyright © 2019 Shimosaka et al. 2019 Shimosaka et al. This content is distributed under the terms of the Creative Commons Attribution 4.0 International license.

### Search for a novel DPCK in T. kodakarensis.

Given that DPCK activity was not observed with the recombinant TK1334 and TK2192 proteins, we searched for a novel DPCK gene that would be nonhomologous or perhaps extremely distantly related to previously identified and characterized DPCKs from eukaryotes and bacteria. From the results of metagenomic analyses, we found that uncharacterized genes classified into arCOG04076 were fused with PPAT in many genomes from uncultured archaea (e.g., AIF21550.1 from group II/III euryarchaeota member SAT1000_05_B04). PPAT catalyzes the adenylyl-transfer reaction from ATP to 4′-phosphopantetheine to generate dephospho-CoA, the step that directly precedes the DPCK reaction in the classical CoA biosynthesis pathway. Given that gene fusion often implies a functional relationship between two genes, we sought to characterize TK1697, the member of arCOG04076 from T. kodakarensis, although this gene is not fused to the predicted PPAT gene (TK2128).

### Production, purification, and characterization of recombinant TK1697 protein.

The TK1697 gene was expressed in E. coli, and the recombinant protein was purified. The sample was subjected to SDS-PAGE, and a single band corresponding to the calculated molecular mass of TK1697 (19,657 Da) was observed (Fig. S1C), indicating that the protein was purified to apparent homogeneity. The purified TK1697 protein eluted as a single peak in gel filtration chromatography and corresponded to a molecular mass of approximately 20.8 kDa. The estimated molecular mass from the amino acid sequence of the TK1697 protein was 19,657 Da, indicating that the TK1697 protein was a monomer in solution.

### DPCK activity of the TK1697 protein.

**FIG 2:**
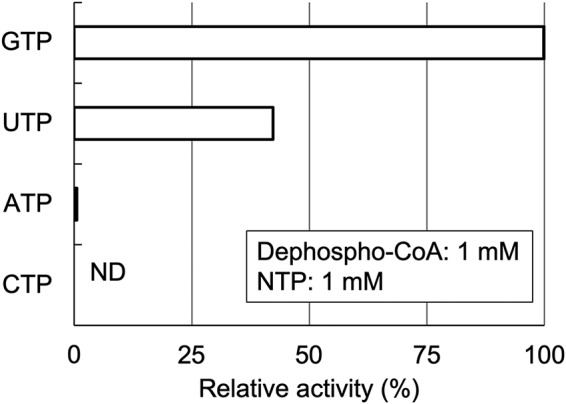
Phosphate donor specificity of the TK1697 protein. The reaction mixture contained 1 mM dephospho-CoA, 1 mM NTP, 5 mM MgCl_2_, and 10 μg ml^−1^ recombinant protein for UTP and GTP or 50 μg ml^−1^ recombinant protein for ATP and CTP in 50 mM Bicine (pH 8.0).

The purified, recombinant TK1697 protein was incubated with ATP and dephospho-CoA. Generation of CoA was observed, and the amount of CoA increased linearly with the reaction time, but the activity was low (Fig. S2). When other nucleotides (UTP, GTP, or CTP) were used as the phosphate donor instead of ATP, the TK1697 protein showed the highest activity with GTP (Fig. 2). These results show that the TK1697 protein possesses a GTP-dependent DPCK activity. When NAD^+^, ADP, AMP, adenosine, or ribose was substituted for dephospho-CoA as phosphate acceptors, no detectable amount of GDP was produced, indicating specificity of the TK1697 protein toward dephospho-CoA.

10.1128/mBio.01146-19.2 FIG S2 Chromatograms of the peak corresponding to CoA after the DPCK reaction with ATP. Aliquots (10 μl) of the reaction mixture were applied to a Cosmosil 5C_18_-PAQ 4.6-mm-inside-diameter by 250-mm column. Compounds were separated with 20 mM sodium phosphate (pH 6.1) at a flow rate of 1.0 ml min^−1^ at 40°C. Absorbance at 254 nm was measured. Line colors indicate reaction times of 1 min (red), 3 min (orange), 5 min (green), 10 min (blue), and 10 min without TK1697 protein (purple). Download FIG S2, TIF file, 0.4 MB. Copyright © 2019 Shimosaka et al. 2019 Shimosaka et al. This content is distributed under the terms of the Creative Commons Attribution 4.0 International license.

**FIG 3:**
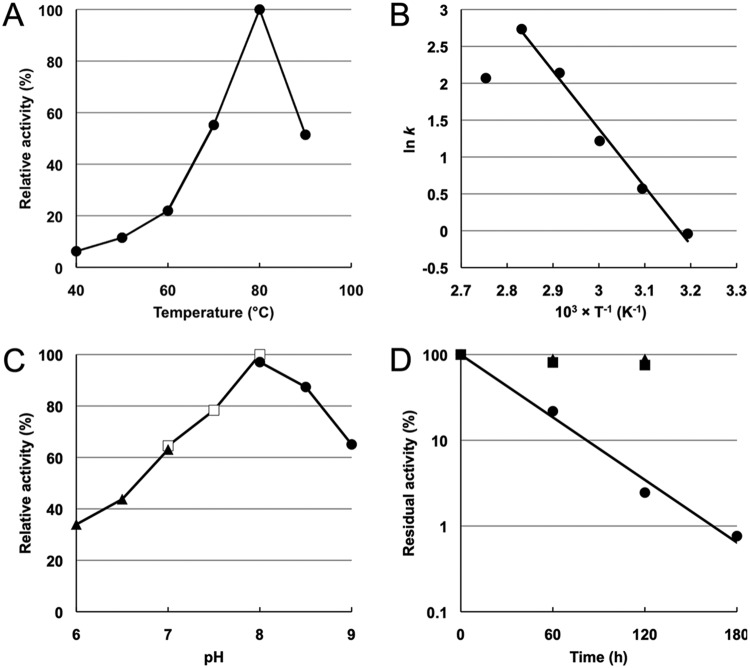
Effect of temperature and pH on the DPCK activity of the TK1697 protein. (A) Effects of temperature on DPCK activity. (B) Arrhenius plot of the data shown in panel A. (C) Effects of pH on DPCK activity. Symbols: closed triangles, MES; open squares, HEPES; closed circles, Bicine. (D) Thermostability of the TK1697 protein. Symbols: squares, 70°C; triangles, 80°C; circles, 90°C.

The effects of temperature and pH on the DPCK activity of the TK1697 protein were examined. Under our assay conditions, the TK1697 protein exhibited highest activity at 80°C (Fig. 3A). From the Arrhenius plot of the data in Fig. 3A, the activation energy of this reaction was calculated as 65.3 kJ mol^−1^ (Fig. 3B). When the DPCK reaction was performed at various pHs, the TK1697 protein exhibited highest activity at pH 8.0 (Fig. 3C). The thermostability of the protein was examined at 70, 80, or 90°C. The TK1697 protein did not lose its activity after 2 h of incubation at 70 or 80°C, and the half-life at 90°C was calculated to be approximately 24 min (Fig. 3D).

### Kinetics of the dephospho-CoA kinase reaction.

**FIG 4:**
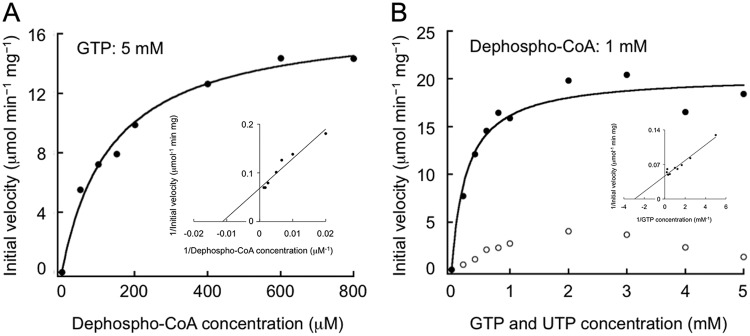
Kinetic examination of the dephospho-CoA kinase reaction. DPCK activity assays were performed with various concentrations of dephospho-CoA with 5 mM GTP (A) or GTP and UTP with 1 mM dephospho-CoA (B). Symbols: closed circles, GTP; open circles, UTP. Measurements were performed at 80°C. Insets show double reciprocal plots of the data.

DPCK activity assays were performed with various concentrations of dephospho-CoA (with 5 mM GTP) and GTP or UTP (with 1 mM dephospho-CoA). The results of the assays with dephospho-CoA and GTP followed Michaelis-Menten kinetics (Fig. 4), and the obtained parameters are indicated in Table 1. The results of assays with UTP did not follow Michaelis-Menten kinetics, and inhibition of the activity was observed at high UTP concentrations (Fig. 4B). These findings are compatible with GTP being the preferred phosphate donor for TK1697. The kinetic measurements indicated a comparatively low substrate affinity (high *K_m_*) for the TK1697 DPCK activity but also high catalytic activity (*k*_cat_).

**TABLE 1:** Kinetic parameters of DPCK toward dephospho-CoA and GTP^*a*^

Source	Substrate	*V*_max_ (μmol min^−1^ mg^−1^)	*K_m_* (mM)	*k*_cat_ (s^−1^)	*k*_cat_/*K_m_* (s^−1^ mM^−1^)
T. kodakarensis	Dephospho-CoA	17.0 ± 0.8	0.14 ± 0.02	5.57	40.4
	GTP	20.4 ± 1.0	0.26 ± 0.06	6.68	25.7
M. tuberculosis	Dephospho-CoA	—	0.035	0.029	0.83
	ATP	—	0.057	0.048	0.84
E. coli	Dephospho-CoA	—	0.14	—	—
	ATP	—	0.74	—	—
E. histolytica (EhDPCK1)	Dephospho-CoA	3.71	0.11	1.48	13.5
	ATP	3.54	0.020	1.41	70.5
E. histolytica (EhDPCK2)	Dephospho-CoA	2.48	0.058	0.96	16.5
	ATP	2.71	0.015	1.05	70

a Reaction temperature for T. kodakarensis DPCK was 80°C. Values for the enzymes from M. tuberculosis, E. coli, and E. histolytica were obtained from previous studies (32, 36, 40). —, not reported.

### Disruption of the TK1697 gene.

To assess the contribution of TK1697 to CoA biosynthesis in T. kodakarensis, we constructed a gene disruption strain and compared its phenotype with that of its host strain, T. kodakarensis KPD1. We initially constructed a strain with almost the entire coding region of TK1697 removed but realized that the resulting deletion affected the expression of the immediate downstream genes TK1696 and TK1695. The disruption plasmid was thus designed to remove a region that corresponds to residues 31 to 210 of the coding region (Fig. S3) and maintain the putative transcription factor B recognition element (BRE)/TATA sequences and the transcription initiation site (21). Transformants were selected on solid medium that included 5-fluoroorotic acid (5-FOA), agmatine (1 mM), and CoA (1 mM). CoA was added given the expectation that the transformant would not grow without CoA, as previously observed in the disruption strains for PoK and PPS genes (8). A number of transformants were examined, and PCR analysis (Fig. S4A) and DNA sequencing of their genomic DNA confirmed the isolation of a transformant with deletion of the TK1697 gene (T. kodakarensis K1697).

10.1128/mBio.01146-19.3 FIG S3 Strategy for TK1697 gene disruption. (A) Overview of the region surrounding TK1697. Gene annotations are as follows: TK1695, small subunit ribosomal protein S27Ae; TK1696, small subunit ribosomal protein S24e; TK1697, dephospho-CoA kinase; TK1698, DNA-directed RNA polymerase subunit E″; TK1699, DNA-directed RNA polymerase subunit E′. The red band indicates the region including the TATA and BRE sequences. The shaded region corresponds to the region removed in the gene disruption strain T. kodakarensis ΔTK1697. (B) The entire coding region of TK1697 is shown. The gray letters correspond to the nucleotides removed in T. kodakarensis ΔTK1697. The BRE sequence and TATA sequences are shown in red and blue, respectively. The predicted initiation codon of TK1696 is underlined and shown in capital letters. Download FIG S3, TIF file, 0.4 MB. Copyright © 2019 Shimosaka et al. 2019 Shimosaka et al. This content is distributed under the terms of the Creative Commons Attribution 4.0 International license.

10.1128/mBio.01146-19.4 FIG S4 PCR analyses of the T. kodakarensis ΔTK1697 (A), ΔTK0517 (B), and ΔTK2128 (C) strains. H, host strain KPD1 (A) or KU216 (B and C); Δ, disruption strain. Inside, primer set that amplifies a region within the coding region of each target gene was used for the PCR; outside, primer set that amplifies from outside the flanking regions of each target gene was used for the PCR. Download FIG S4, TIF file, 2.7 MB. Copyright © 2019 Shimosaka et al. 2019 Shimosaka et al. This content is distributed under the terms of the Creative Commons Attribution 4.0 International license.

**FIG 5:**
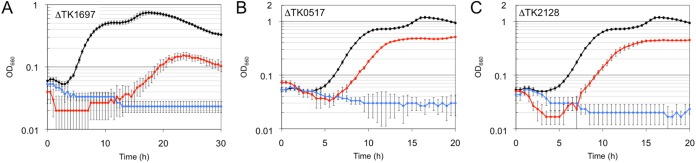
Growth characteristics of T. kodakarensis ΔTK1697 (A), ΔTK0517 (B), and ΔTK2128 (C) and their host strains. Cells were cultivated in ASW-YT-pyruvate-agmatine medium (A) or ASW-YT-pyruvate medium (B and C) at 85°C. Symbols: black circles, KPD1 (A) or KU216 (B and C); blue circles, disruption strains; red circles, disruption strains grown in medium supplemented with 1 mM CoA. Error bars represent the standard deviations from three independent experiments.

T. kodakarensis K1697 (ΔTK1697) was inoculated in ASW-YT-pyruvate-agmatine medium, but no growth was observed (Fig. 5A). When 1 mM CoA was added to the medium, the growth defect was partially complemented, with lower growth rate and less cell yield than the host strain KPD1 (Fig. 5A). The results indicate that TK1697 contributes to CoA biosynthesis in T. kodakarensis. In order to examine whether the addition of higher concentrations of CoA would better complement the growth defects of ΔTK1697, we grew the disruption strain in the presence of 0, 0.1, 0.5, 1, 2, and 5 mM CoA (Fig. S5A). With 0.1 mM CoA, ΔTK1697 did not display growth. In the presence of 0.5 mM CoA, we observed growth of ΔTK1697 but with a growth rate lower than that observed with 1 mM CoA. Growth with 2 mM CoA was similar to that observed with 1 mM CoA, but growth initiation was slightly delayed. The presence of 5 mM CoA completely abolished growth. The growth properties with 0, 0.1, 0.5, 1, and 2 mM CoA indicate that CoA stimulates growth in a concentration-dependent manner, and its effect saturates at around 1 mM CoA. Growth properties with 1, 2, and 5 mM CoA indicate that excess concentrations of CoA inhibit growth. This inhibitory effect was also observed in the host strain KPD1. As in ΔTK1697, 2 mM CoA resulted in a slight delay in initiation of growth, and 5 mM CoA resulted in a lower growth rate and lower cell yield (Fig. S5B). Although transporters involved in the uptake of compounds related to CoA biosynthesis or degradation have been reported, transporters responsible for the uptake of intact CoA have not been identified, suggesting their absence in most microorganisms (2, 3, 22–24). We presume that this is also the case in T. kodakarensis and that the direct uptake of intact CoA, which is necessary for compensating the absence of DPCK, is inefficient in this organism, resulting in only partial complementation with exogenous CoA.

10.1128/mBio.01146-19.5 FIG S5 Growth characteristics of T. kodakarensis ΔTK1697 (A) and KPD1 (B) in the presence or absence of various concentrations of CoA. Cells were cultivated in ASW-YT-pyruvate-agmatine medium at 85°C. Symbols: black circles, no CoA; purple circles, 0.1 mM CoA; red circles, 0.5 mM CoA; blue circles, 1 mM CoA; green circles, 2 mM CoA; orange circles, 5 mM CoA. Error bars represent the standard deviations from three independent experiments. Download FIG S5, PDF file, 0.4 MB. Copyright © 2019 Shimosaka et al. 2019 Shimosaka et al. This content is distributed under the terms of the Creative Commons Attribution 4.0 International license.

### In *trans* TK1697 gene expression.

**FIG 6:**
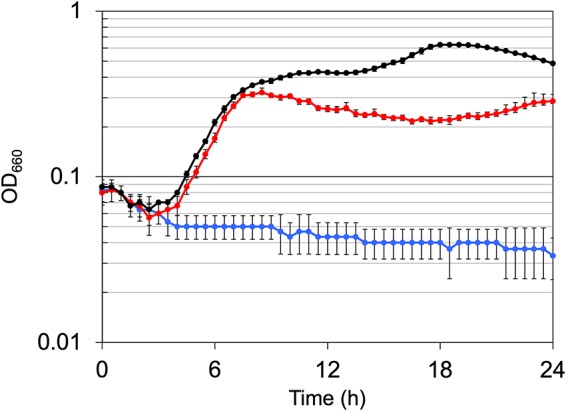
Growth characteristics of T. kodakarensis ΔTK1697 (Δ*pyrF* Δ*pdaD* ΔTK1697) transformed with the wild-type TK1697 gene. Cells were cultivated in ASW-YT-pyruvate medium at 85°C. Symbols: black circles, the host strain KPD1 (Δ*pyrF* Δ*pdaD*) transformed with pRPG03-f; blue circles, disruption strain transformed with pRPG03-f; red circles, disruption strain transformed with pRPETK1697. Error bars represent the standard deviations from three independent experiments.

To examine whether in *trans* TK1697 gene expression complements the growth defect of the ΔTK1697 strain, a TK1697 gene expression plasmid, pRPETK1697, was constructed. The T. kodakarensis strain K1697 was transformed with the empty plasmid pRPG03-f or pRPETK1697. Only cells that harbor pRPG03-f or pRPETK1697 can grow in the absence of agmatine. Transformants were isolated on ASW-YT-S^0^ solid medium supplemented with 1 mM CoA. The disruption strains transformed with pRPG03-f or pRPETK1697 were cultivated in ASW-YT-pyruvate medium (Fig. 6). No growth was observed for the strain harboring pRPG03-f. In contrast, in the strain transformed with pRPETK1697, the growth defect was almost fully complemented (Fig. 6).

### Evolutionary relationship between archaeal DPCKs and thiamine pyrophosphokinases and site-directed mutagenesis of predicted catalytic residues.

Iterative PSI-BLAST search of the NCBI protein sequence database for putative homologs of TK1697 failed to retrieve any sequences apart from the arCOG04076 members that are present in nearly all archaea (25) (Fig. S6A). However, searches using HHpred initiated with protein sequences from arCOG04076 revealed a moderate similarity (probability of 50 to 60%) between the archaeal proteins of arCOG04076 and bacterial thiamine pyrophosphokinases (Fig. S6B). Examination of the multiple alignment between the arCOG04076 proteins and thiamine pyrophosphokinases shows conservation of the key secondary structural elements of this distinct fold and two of the four amino acid residues that comprise the catalytic site based on comparison with experimentally and structurally characterized thiamine pyrophosphokinase from mouse (PDB 2F17 [Fig. S6]) (26).

10.1128/mBio.01146-19.6 FIG S6 Multiple alignment of novel archaeon-specific DPCK family and comparison with thiamine pyrophosphokinase, a distant homolog. (A) Multiple alignment of 163 sequences assigned to arCOG04076 with predicted secondary structure and 95% amino acid identity consensus lines shown underneath the alignment. Each sequence is denoted by the protein accession number or locus tag for the sequence from Thermococcus kodakarensis. (B) Comparison of archaeon-specific DPCK family and thiamine pyrophosphokinase ThiN family based on HHpred alignments (protein YP_004457895.1 and PDB accession no. 3MEL, thiamine pyrophosphokinase from Enterococcus faecalis, are a query and subject, respectively). Only the region alignable by HHpred is shown. Catalytic residues shown underneath PDB 2F17 (mouse thiamine pyrophosphokinase) sequences are shown by red asterisks according to the data from the work of Liu et al. (J. Y. Liu, D. E. Timm, and T. D. Hurley, J Biol Chem 281:6601–6607, 2006, https://doi.org/10.1074/jbc.M510951200). Common conserved aspartate residues are shown in red. Abbreviations: SS, secondary structure. Download FIG S6, PDF file, 0.2 MB. Copyright © 2019 Shimosaka et al. 2019 Shimosaka et al. This content is distributed under the terms of the Creative Commons Attribution 4.0 International license.

**FIG 7:**
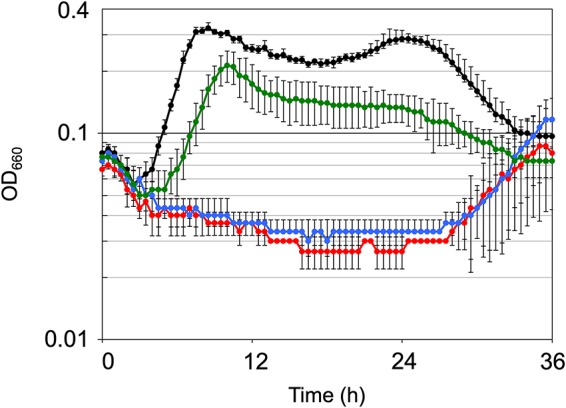
Growth characteristics of T. kodakarensis ΔTK1697 (Δ*pyrF* Δ*pdaD* ΔTK1697) transformed with pRPETK1697(D48A), pRPETK1697(D67A), or pRPETK1697(D125A). Cells were cultivated in ASW-YT-pyruvate medium at 85°C. Symbols: black circles, disruption strain transformed with pRPETK1697; red circles, disruption strain transformed with pRPETK1697(D48A); blue circles, disruption strain transformed with pRPETK1697(D67A); green circles, disruption strain transformed with pRPETK1697(D125A). Error bars represent the standard deviations from three independent experiments.

We hypothesized that negatively charged amino acid residues, Asp48, Asp67, and Asp125 of TK1697, which are conserved in the arCOG04076 sequences, with the first two being also conserved in the catalytic site of thiamine pyrophosphokinases, could directly participate in the archaeal DPCK catalysis (Fig. S6). Mutants with Ala replacing each of these conserved Asp residues were constructed and individually incorporated into pRPG03-f, resulting in the plasmids pRPETK1697(D48A), pRPETK1697(D67A), and pRPETK1697(D125A) that were introduced into T. kodakarensis K1697. Disruption strains transformed with each mutant TK1697 as well as the wild-type expression vector were cultivated in ASW-YT-pyruvate medium. The D48A and D67A mutants showed a dramatic reduction in growth rates compared to the disruption strain transformed with the wild-type TK1697 (Fig. 7). The D125A mutant showed a reduction in growth rates compared to the wild-type TK1697 but showed a better growth rate than the D48A and D67A mutants. These results further support the conclusion that TK1697 and its orthologs are archaeal DPCKs and also suggest conservation of the catalytic site between this novel DPCK and thiamine pyrophosphokinases.

### Disruption of TK0517 and TK2128 genes.

In T. kodakarensis, five genes (KPHMT, KPR, GDC, PoK, and PPS) involved in CoA biosynthesis have been previously characterized (4–8), and in this study a novel DPCK was identified and characterized. In order to experimentally validate the genes responsible for the remaining steps of CoA biosynthesis in T. kodakarensis, namely, those catalyzed by PPCS, PPCDC, and PPAT, the genes predicted to encode bifunctional PPCS-PPCDC (TK0517) and PPAT (TK2128) were genetically examined. Gene disruption strains for TK0517 and TK2128 were constructed by the same procedure that was used to disrupt the TK1697 gene. PCR analysis and DNA sequencing of genomic DNA confirmed the isolation of transformants with deletions of each target gene (Fig. S4B and C).

The TK0517 gene disruption strain (ΔTK0517) and the TK2128 gene disruption strain (ΔTK2128) were cultivated in ASW-YT-pyruvate medium. Growth was not observed in the absence of exogenous CoA (Fig. 5B and C). When 1 mM CoA was added to the medium, although with lower growth rates and lower cell yields than the host strain, the mutant strains displayed growth (Fig. 5B and C), indicating that these genes are involved in the biosynthesis of CoA in T. kodakarensis. We noticed that the addition of CoA better complemented the growth defects of ΔTK0517 and ΔTK2128 than that of ΔTK1697. This may be related to where the individual reactions are located within the biosynthesis pathway. DPCK catalyzes the final reaction of the pathway, and as described above, its absence would require the direct uptake of intact CoA for complementation. The ΔTK2128 strain without PPAT and the ΔTK0157 strain without PPCS-PPCDC can be complemented with dephospho-CoA and dephospho-CoA/4′-phosphopantetheine, respectively, in addition to CoA. This would enable the latter two strains to utilize a wider range of compounds that might be formed by the degradation of CoA in the medium and taken up.

## DISCUSSION

Biochemical analyses in this study show that the TK1697 protein from T. kodakarensis possesses a GTP-dependent DPCK activity. This conclusion is further supported by the observation of CoA auxotrophy in TK1697 knockouts and by site-directed mutagenesis of putative catalytic amino acid residues. The products of TK1334 and TK2192 that encode homologs of bacterial and eukaryotic DPCK and, accordingly, have been annotated as putative DPCK lacked DPCK activity. These findings suggest that TK1697 is the only DPCK in this archaeon (Tk-DPCK).

Orthologs of TK1697, members of arCOG04076, are represented in nearly all archaea, with the exception of *Nanoarchaeota* and some other members of the DPANN superphylum that are known or predicted to be parasites of other archaea and apparently depend on their hosts for most metabolites and coenzymes. Amino acid sequences of the arCOG04076 proteins show no significant similarity to those of previously identified DPCKs from bacteria and eukaryotes. However, a sensitive HHpred search showed that members of arCOG04076 are distantly related to bacterial and eukaryotic thiamine pyrophosphokinases. Because structures of several thiamine pyrophosphokinases have been solved, this homology allows one to predict the structural fold of the archaeal DPCK and the catalytic residues, with the latter prediction validated by site-directed mutagenesis. Structurally, thiamine pyrophosphokinases and, by inference, archaeal DPCK represent a highly derived variant of the Rossmann fold that is characteristic of numerous metabolic enzymes (27) but distinct from the P-loop fold of the bacterial and eukaryotic DPCKs (28).

From the evolutionary standpoint, the novel archaeal DPCK and the previously studied bacterial and eukaryotic DPCKs represent a typical pair of analogous enzymes, that is, enzymes that are structurally and evolutionarily unrelated but convergently evolve to catalyze the same reaction (29, 30). Notably, analogous enzymes include many that adopt the Rossmann fold as well as many kinases (30). Thus, the convergent evolution of the two nonhomologous versions of DPCK represents a general pattern of enzyme evolution. The distribution of TK1697 homologs in *Euryarchaeota* is wider than those observed for PoK and PPS, which are also archaeon-specific enzymes but are missing in *Thermoplasmatales*. Thus, the existence of additional pairs of analogous enzymes in the CoA biosynthesis pathway can be predicted. Furthermore, the functions of TK1334 and TK2192 (both from arCOG01045), the two P-loop fold archaeal proteins that are homologous to bacterial and eukaryotic DPCKs, remain unclear. Nevertheless, the presence of members of arCOG01045, to which both of these proteins belong, in nearly all archaea suggests that at least some of these proteins perform essential functions. Identification of the biochemical activities and physiological functions of these predicted kinases can be expected to reveal new pairs of analogous enzymes.

An unexpected feature of the archaeal DPCK is the strong preference for GTP as the phosphate donor. All previously characterized DPCKs are considered to be ATP dependent, although direct experimental evidence is limited. In bacteria, DPCK enzymes from Thermus thermophilus HB8 (31), Mycobacterium tuberculosis (32–34), Streptomyces peucetius ATCC 27952 (35), and E. coli K-12 (36) have been characterized. However, in the cases of the enzymes from T. thermophilus, *S. peucetius*, and E. coli, phosphate donors other than ATP have not been examined. For DPCK from M. tuberculosis, various nucleoside triphosphates (NTPs) and deoxynucleoside triphosphates (dNTPs) (ATP, GTP, CTP, ADP, dATP, and dGTP) were used as phosphate donors, and DPCK activity was observed only with ATP and dATP, providing experimental evidence that the enzyme is ATP dependent. In eukaryotes, DPCK proteins are generally fused with PPAT proteins, and the bifunctional enzymes are designated CoA synthases. The DPCK activity of CoA synthase from human cells has been characterized, but ATP was the only phosphate donor examined (37–39). Entamoeba histolytica has two standalone DPCK proteins (40). ATP, TTP, GTP, CTP, and UTP were used as phosphate donors, and both enzymes showed highest activity with ATP. Thus, although further experimental validation will be needed for bacterial and eukaryotic enzymes, the T. kodakarensis DPCK shows a stark difference from the bacterial M. tuberculosis and eukaryotic E. histolytica enzymes in terms of phosphate donor specificity.

Kinetic parameters of DPCK have been reported for M. tuberculosis, E. coli K-12, and E. histolytica (Table 1) (32, 36, 40). The T. kodakarensis DPCK shows a relatively low affinity for both substrates (high *K_m_*) and, in this respect, is more similar to the E. coli DPCK than to that of M. tuberculosis. However, due to the high *k*_cat_ values, the archaeal DPCK has a much higher *k*_cat_/*K_m_* ratio than any of the characterized bacterial enzymes, i.e., is a much more catalytically efficient enzyme. The E. histolytica enzymes display relatively high affinity for both substrates, and their *k*_cat_/*K_m_* ratios are comparable with that of T. kodakarensis DPCK.

In addition to the identification of the archaeal DPCK, we also demonstrated the requirement of the predicted bifunctional PPCS-PPCDC (TK0517) and PPAT (TK2128) for CoA biosynthesis. Indeed, gene disruption strains for both these genes display CoA auxotrophy. Taken together with the results of previous studies (4–8), all 8 genes involved in the biosynthesis of CoA from 2-oxoisovalerate (Fig. 1), as well as the gene encoding aspartate decarboxylase, the enzyme that supplies β-alanine, are now identified in T. kodakarensis. Compared with the pathways in bacteria and eukaryotes, the conversion from pantoate to 4′-phosphopantothenate follows different chemistry (PoK-PPS versus PS-PanK), with phosphorylation preceding condensation in archaea (8). The enzyme responsible for β-alanine biosynthesis in archaea is structurally distinct from those found in bacteria and eukaryotes (7). As shown here, DPCK is also structurally distinct from its counterparts in bacteria and eukaryotes and utilizes GTP as the phosphate donor. Regulation of CoA biosynthesis also differs, with feedback regulation targeting KPR in archaea, in contrast to PanK in bacteria and eukaryotes (5).

Thus, the results of the present study not only fill a major gap in our understanding of archaeal metabolism but also expand our understanding of the role of convergence in the evolution of central metabolism in different domains of life.

## MATERIALS AND METHODS

### Strains and growth conditions.

E. coli strains DH5α (TaKaRa, Ohtsu, Japan) and BL21-CodonPlus(DE3)-RIL (Agilent Technologies, Santa Clara, CA) were cultivated at 37°C in lysogeny broth (LB) medium containing 100 mg liter^−1^ ampicillin. T. kodakarensis strains were cultivated at 70°C or 85°C in a nutrient-rich medium (ASW-YT-S^0^ or ASW-YT-pyruvate) or a minimal medium (ASW-AA-S^0^) under anaerobic conditions. ASW-YT-S^0^ medium consisted of 0.8× artificial seawater (ASW) (41), 5.0 g liter^−1^ yeast extract, 5.0 g liter^−1^ tryptone, 0.8 mg liter^−1^ resazurin, and 2.0 g liter^−1^ elemental sulfur. In ASW-YT-pyruvate medium, elemental sulfur was replaced with 5.0 g liter^−1^ sodium pyruvate. ASW-AA-S^0^ medium consisted of 0.8× ASW, a mixture of 20 amino acids, modified Wolfe’s trace minerals, a vitamin mixture, and 2.0 g liter^−1^ elemental sulfur (12, 37). Prior to inoculation, Na_2_S was added to the medium until it became colorless. For solid medium, elemental sulfur and Na_2_S were replaced with 2 ml liter^−1^ of a polysulfide solution (10 g Na_2_S·9H_2_O and 3 g sulfur flowers in 15 ml H_2_O), and 10 g liter^−1^ Gelrite was added. Unless mentioned otherwise, chemicals were purchased from Wako Pure Chemicals (Osaka, Japan) or Nacalai Tesque (Kyoto, Japan).

### Production and purification of the TK1334, TK1697, and TK2192 recombinant proteins.

The TK1334 gene was amplified from genomic DNA of T. kodakarensis KOD1 using the primer set TK1334F/TK1334R (Table 2). The amplified fragment was inserted into the NdeI and BamHI sites of pET21a(+) (Merck KGaA, Darmstadt, Germany). The TK1697 and TK2192 genes were amplified using the primer sets TK1697F/TK1697R1 and TK2192F/TK2192R, respectively. Amplified fragments were individually inserted into pET21a(+) at the NdeI and EcoRI sites. After sequence confirmation, the plasmids were introduced into E. coli BL21-CodonPlus(DE3)-RIL. Transformants were grown in LB medium until the optical density at 660 nm reached 0.4. Isopropyl-1-thio-β-d-galactopyranoside was added to a final concentration of 0.1 mM to induce gene expression, and cells were cultivated for a further 4 h. For cells harboring TK1334 and TK2192, cells were harvested by centrifugation (4°C, 5,000 × *g*, 15 min) and suspended in 50 mM Tris-HCl (pH 7.5) with 150 mM NaCl. After centrifugation (4°C, 5,000 × *g*, 15 min), cells were suspended with 50 mM Tris-HCl (pH 7.5) and disrupted by sonication. After centrifugation (4°C, 5,000 × *g*, 15 min), the soluble cell extract was incubated at 90°C for 10 min. Cells harboring TK1697 were harvested and disrupted in the same manner, but the buffer was 50 mM sodium phosphate (pH 4.5), and heat treatment was at 70°C for 10 min. All protein samples were subjected to centrifugation (4°C, 5,000 × *g*, 15 min), and the supernatants were filtered through an 0.2-μm New Steradisc sterilized filter (Kurabo, Osaka, Japan). TK1334 and TK2192 samples were applied to a Resource Q 6-ml anion-exchange chromatography column (GE Healthcare, Little Chalfont, Buckinghamshire, United Kingdom) and eluted with a linear gradient of NaCl (0 to 1.0 M) in 50 mM Tris-HCl (pH 7.5) at a flow rate of 2.0 ml min^−1^. Relevant fractions were concentrated with an Amicon Ultra-4 10 K centrifugal filter (Millipore, Billerica, MA), filtered, and applied to a Superdex 200 Increase 10/300 GL gel filtration column (GE Healthcare). Proteins were eluted with 50 mM Tris-HCl (pH 7.5) including 150 mM NaCl at a flow rate of 0.4 ml min^−1^. TK1697 sample was applied to a HiTrap SP HP cation-exchange chromatography column (GE Healthcare) and eluted with a linear gradient of NaCl (0 to 1.0 M) in 50 mM sodium phosphate (pH 4.5) at a flow rate of 5.0 ml min^−1^. Relevant fractions were concentrated, filtered, and applied to a Superdex 200 Increase 10/300 GL gel filtration column. Proteins were eluted with 50 mM sodium phosphate (pH 4.5) including 150 mM NaCl at a flow rate of 0.4 ml min^−1^. For examining the molecular mass of proteins, RNase A (13.7 kDa), carbonic anhydrase (29 kDa), conalbumin (75 kDa), and ferritin (440 kDa) (GE Healthcare) were used as standards. All chromatography procedures were performed using an Äkta Explorer system. Protein concentration was determined with the Protein Assay system (Bio-Rad, Hercules, CA) using bovine serum albumin as a standard. The samples were subjected to sodium dodecyl sulfate-polyacrylamide gel electrophoresis (SDS-PAGE), and the gel was stained with Coomassie brilliant blue.

**TABLE 2:** Sequences of primers used in this study

Primer name	Primer sequence
TK1334F	5′-GGGCCATATGATAGTCATAGTCACTGGAATGC-3′
TK1334R	5′-AAAAGGATCCTCAAAGCTTCGAGATAATTTCAT-3′
TK1697F	5′-AAAGGATCCCATATGAAAATGTTCTTCAGGCTT-3′
TK1697R1	5′-AAAGAATTCTTAATCTCCATCACGAACCA-3′
TK1697R2	5′-AAAGTCGACTTAATCTCCATCACGAACCA-3′
TK2192F	5′-TGGCGCATATGGAGGCGGGGAAGATGATAATAGGCG-3′
TK2192R	5′-ATGAGAATTCTCATCCCATTACCTCCGAAATTATCTCCT-3′
DTK0517F	5′-CATCGGCGAGAACGACCGCT-3′
DTK0517R	5′-CGTGCTCCTGACTGTGTGAAAGAG-3′
DTK1697F	5′-CTGGAAGGACATAGATGACGTTCCAAAGGC-3′
DTK1697R	5′-ATCCGCCATGGACGCCCGACAGGATAAGCC-3′
DTK2128F	5′-GCTTGTCGGTTGCCAGAACAC-3′
DTK2128R	5′-CCTTCGGGCACTATGCGGTG-3′
DTK0517invF	5′-CCCGGTTTTATCTTTTATCGCTTATTC-3′
DTK0517invR	5′-CTGAATCACCGTAGGTAGTTAGAAAAAGGG-3′
DTK1697invF1	5′-ATGGAGATTAAGGTTACCGA-3′
DTK1697invR1	5′-TTTCACCTTACCCTGATGGC-3′
DTK1697invF2	5′-AAGAGAAAAGAATACTCCCCCGA-3′
DTK1697invR2	5′-TTCTCTAGTAAGCCTGAAGAACATTTT-3′
DTK2128invF	5′-GCCCAAAGGTGATAACACTA-3′
DTK2128invR	5′-GGTCCTACTTACTCATGCAGACCAATAA-3′
TK1697(D48A)F	5′-GCCGTCGTCACGGAGAACGTCCT-3′
TK1697(D48A)R	5′-CCCAACAGTGACAACGGGGTGTTT-3′
TK1697(D67A)F	5′-GCCCTCAAGACGAAGAGAAAAGAATAC-3′
TK1697(D67A)R	5′-GTAAAGGGCAATTATTGGTTTGACGC-3′
TK1697(D125A)F	5′-GCCTTGGCGGCGATCCCGG-3′
TK1697(D125A)R	5′-CTCCTCCCCGCTGACCAGGATGT-3′

### Examination of dephospho-CoA kinase activity of the recombinant proteins.

DPCK activity was measured at 80°C. The reaction mixture contained 1 mM 3′-dephospho-coenzyme A (Sigma-Aldrich, St. Louis, MO), 5 mM GTP, 5 mM MgCl_2_, 300 mM KCl, and 10 μg ml^−1^ recombinant protein in 50 mM *N*,*N*-bis(2-hydroxyethyl)glycine (Bicine) (pH 8.0). After preincubation of the other components, the reaction was initiated by the addition of dephospho-CoA and GTP. The reaction mixture was cooled on ice to stop the reaction, and proteins were removed with an Amicon Ultra-0.5 centrifugal filter unit with an Ultracel-10 membrane (Millipore). An aliquot (10 μl) of filtered solution was applied to a Cosmosil 5C_18_-PAQ 250- by 4.60-mm column (Nacalai Tesque) using a Nexera X2 system (Shimadzu, Kyoto, Japan). Compounds were separated with 20 mM sodium phosphate buffer (pH 6.1) at a flow rate of 1.0 ml min^−1^ at 40°C and detected by absorbance at 254 nm. DPCK activity was measured by monitoring the rate of increase in absorption of CoA. Modifications of this method are described when applied.

### Substrate specificity of dephospho-CoA kinase.

The phosphate donor specificity of DPCK was examined at 70°C. The reaction mixture contained 1 mM dephospho-CoA, 1 mM nucleoside triphosphate (NTP; ATP, UTP, GTP, or CTP), 5 mM MgCl_2_, and 10 μg ml^−1^ recombinant protein for UTP and GTP or 50 μg ml^−1^ recombinant protein for ATP and CTP in 50 mM Bicine (pH 8.0). The phosphate acceptor specificity of DPCK was examined toward NAD^+^, ADP, AMP, adenosine, and ribose at concentrations of 1 mM with 5 mM GTP as the phosphate donor. Kinase activity was measured by monitoring the rate of increase in absorption of GDP. When examining the effects of potassium cations on activity, the reaction mixture contained 1 mM dephospho-CoA, 1 mM GTP, 5 mM MgCl_2_, and 10 μg ml^−1^ recombinant protein in 50 mM Bicine (pH 8.0) with various concentrations of KCl.

### Effects of temperature and pH.

For examining thermostability, the TK1697 protein (0.1 mg ml^−1^) in 50 mM sodium phosphate buffer (pH 4.5) was incubated for various periods of time at 70°C, 80°C, or 90°C. After incubation, the mixture was cooled on ice and residual DPCK activity was measured at 70°C. For examining the effects of temperature on activity, DPCK reactions were performed at various temperatures in 50 mM Bicine (pH 8.0). Effects of pH on activity were examined at 80°C at various pH values using the following 50 mM buffers: 2-morpholineethanesulfonic acid (MES) (pH 5.5 to 7.0), HEPES (pH 7.0 to 8.0), Bicine (pH 8.0 to 9.0), and 2-aminoethanesulfonic acid (CHES) (pH 9.0 to 10.0). For these assays, the reaction mixture contained 1 mM dephospho-CoA, 5 mM GTP, 5 mM MgCl_2_, 300 mM KCl, and 10 μg ml^−1^ recombinant protein.

### Kinetic examination of the dephospho-CoA kinase reaction.

Activity measurements were performed with various concentrations of dephospho-CoA (with 5 mM GTP) or GTP or UTP (with 1 mM dephospho-CoA). Kinetic parameters were calculated with IGOR Pro v. 5.03 (Wave-Metrics, Lake Oswego, OR).

### Gene disruption of TK0517, TK1697, and TK2128.

Gene disruption plasmids for TK0517, TK1697, and TK2128 were constructed by amplifying the individual genes along with 1 kbp of their 5′- and 3′-flanking regions using the primer set DTK0517F/DTK0517R, DTK1697F/DTK1697R, or DTK2128F/DTK2128R, respectively. Amplified fragments were inserted into pUD3, which contains the *pyrF* marker gene cassette inserted into the ApaI site of pUC118. Inverse PCR was performed with the primer set DTK0517invF/DTK0517invR, DTK1697invF1/DTK1697invR1 and DTK1697invF2/DTK1697invR2, or DTK2128invF/DTK2128invR, respectively, followed by self-ligation.

T. kodakarensis strain KU216 (Δ*pyrF*), which shows uracil auxotrophy, was used as the host strain for gene disruption. KU216 was cultivated in ASW-YT-S^0^ medium for 12 h at 85°C. Cells were harvested, suspended in 200 μl of 0.8× ASW, and incubated on ice for 30 min. After addition of 3.0 μg of the gene disruption plasmid and further incubation on ice for 1 h, cells were heated at 85°C for 45 s. Cells were then cultivated twice in ASW-AA-S^0^ medium without uracil for 72 h at 85°C to enrich cells that had undergone single-crossover insertion. Cells were then spread onto solid ASW-YT-S^0^ medium supplemented with 10 g liter^−1^ of 5-FOA, 60 mM NaOH, and 1 mM CoA and grown for 2 days at 70°C. Only cells that have undergone a second recombination that removes the *pyrF* gene are resistant to 5-FOA. Transformants were isolated and cultivated in ASW-YT-S^0^ medium supplemented with 1 mM CoA, and genotypes were analyzed by PCR. Strains with the genotypes Δ*pyrF* ΔTK0517, Δ*pyrF* ΔTK1697, and Δ*pyrF* ΔTK2128 were designated T. kodakarensis K0517, K1697_0, and K2128, respectively. TK1697 was also disrupted using the host strain T. kodakarensis KPD1 (Δ*pyrF* Δ*pdaD*), which shows uracil and agmatine auxotrophy. TK1697 gene disruption was carried out with the same methods described above, but 1 mM agmatine was added to the medium when necessary. The triple gene disruption strain (Δ*pyrF* Δ*pdaD* ΔTK1697) was designated T. kodakarensis K1697_1. We, however, later realized that a putative BRE and TATA box for the downstream ribosomal protein genes were also deleted in K1697_0 and K1697_1. As this would affect the growth of these strains, we reconstructed a new gene disruption strain using K1697_1 as the host strain and the gene disruption plasmid produced with the primer set DTK1697invF2/DTK1697invR2. Gene disruption was carried out with the same methods used to construct K1697_1. The new gene disruption strain was designated T. kodakarensis K1697. In K1697, only the region from nucleotide 31 to nucleotide 210 was removed, and the BRE and TATA box sequences for transcription of TK1696 and TK1695 were retained. Details of the strategy and sequences are illustrated in Fig. S3 in the supplemental material.

### In *trans* expression of TK1697 and its mutant genes.

The TK1697 gene was amplified using the primer set TK1697F/TK1697R2. The amplified fragment was inserted in the NdeI and SalI sites of a T. kodakarensis-E. coli shuttle vector, pRPG03-f (K. Yoshida, T. Kanai, and H. Atomi, unpublished data), and designated pRPETK1697. pRPG03-f is based on pUC118 but with a replication initiator (rep74) from pLC64 (15), a pyruvoyl-dependent arginine decarboxylase gene (*pdaD*) from Pyrococcus furiosus, and a multicloning site between a promoter for the cell surface glycoprotein gene (TK0895) and a terminator for chitinase (TK1765) from T. kodakarensis.

T. kodakarensis K1697 was cultivated in ASW-YT-S^0^ medium supplemented with 1 mM agmatine and 1 mM CoA for 12 h at 85°C. Cells were transformed with pRPG03-f or pRPETK1697 with methods described above. Cells were spread onto ASW-YT-S^0^ medium supplemented with 1 mM CoA. Only cells harboring pRPG03-f or pRPETK1697 can grow in the absence of agmatine. After cultivation for 2 days at 85°C, transformants were isolated and cultivated in ASW-YT-S^0^ medium supplemented with 1 mM CoA. Mutations leading to D48A, D67A, or D125A variants of the TK1697 protein were incorporated into the pRPETK1697 plasmid by inverse PCRs with the primer sets TK1697(D48A)F/TK1697(D48A)R, TK1697(D67A)F/TK1697(D67A)R, or TK1697(D125A)F/TK1697(D125A)R, respectively. The PCR products were treated with DpnI and self-ligated. Introduction of mutations was confirmed, and the plasmids were designated pRPETK1697(D48A), pRPETK1697(D67A), and pRPETK1697(D125A), respectively. Plasmids were introduced into T. kodakarensis K1697 with the same methods used for pRPETK1697.

### Computational sequence analysis.

The PSI-BLAST program (42), with a cutoff E value of 0.001 and with composition-based statistics and low-complexity filtering turned off, was used to search for similar sequences in the NCBI nonredundant (NR) database. HHpred search against CDD and PDB databases with default parameters was used to identify remote homologs and distinct domains in multidomain proteins (43). Multiple sequence alignments were constructed using MUSCLE (44). Protein secondary structure was predicted using Jpred4 (45).
