# Supplementary figures and images for: Identification of Dephospho-Coenzyme A (Dephospho-CoA) Kinase in Thermococcus kodakarensis and Elucidation of the Entire CoA Biosynthesis Pathway in Archaea
Source: mBio. 2019 Jul 23;10(4):e01146-19. doi: 10.1128/mBio.01146-19 (PMC6650551; doi:10.1128/mBio.01146-19)

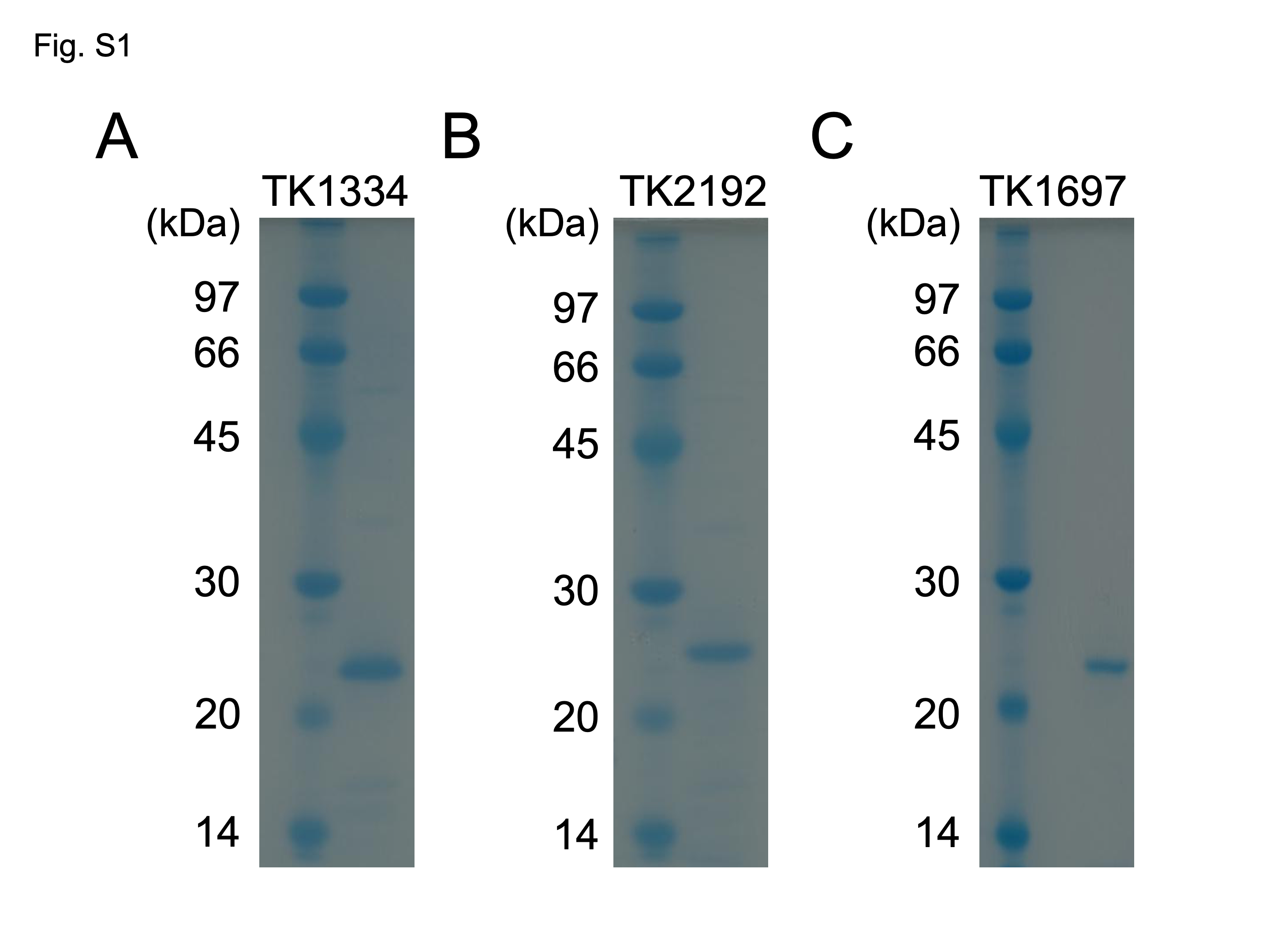

Supplement: FIG S1 [file mBio.01146-19-sf001.tif]

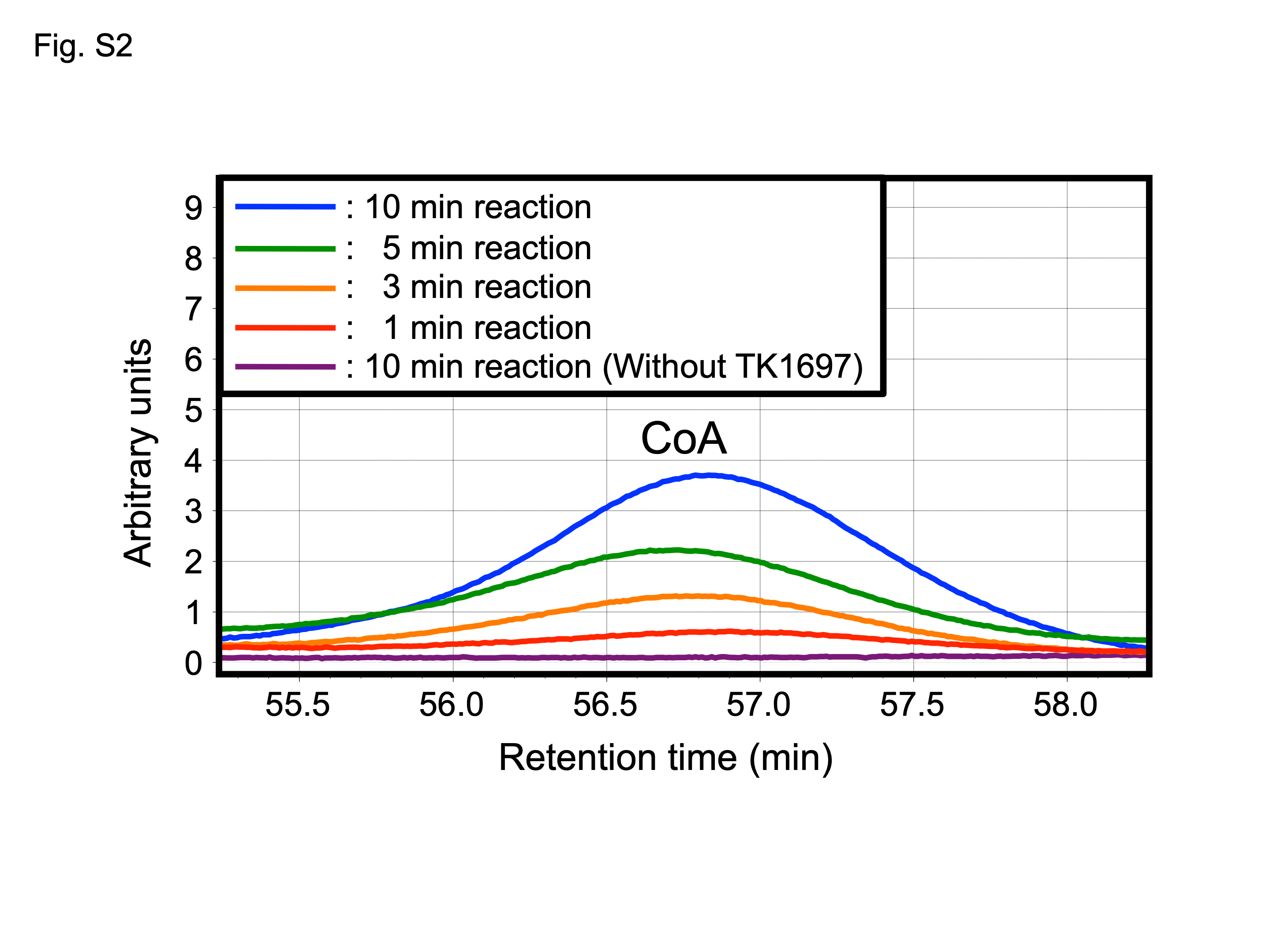

Supplement: FIG S2 [file mBio.01146-19-sf002.tif]

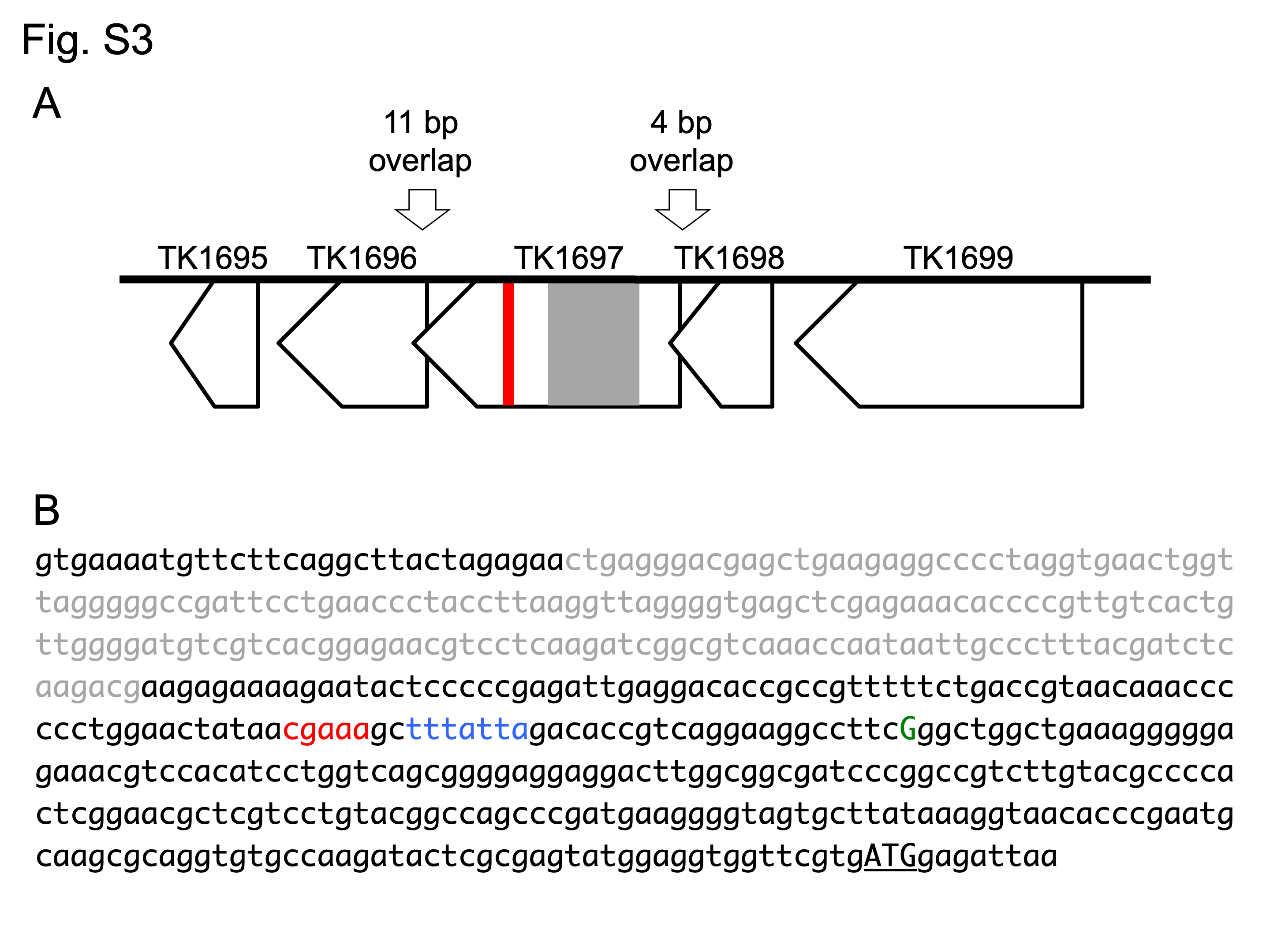

Supplement: FIG S3 [file mBio.01146-19-sf003.tif]

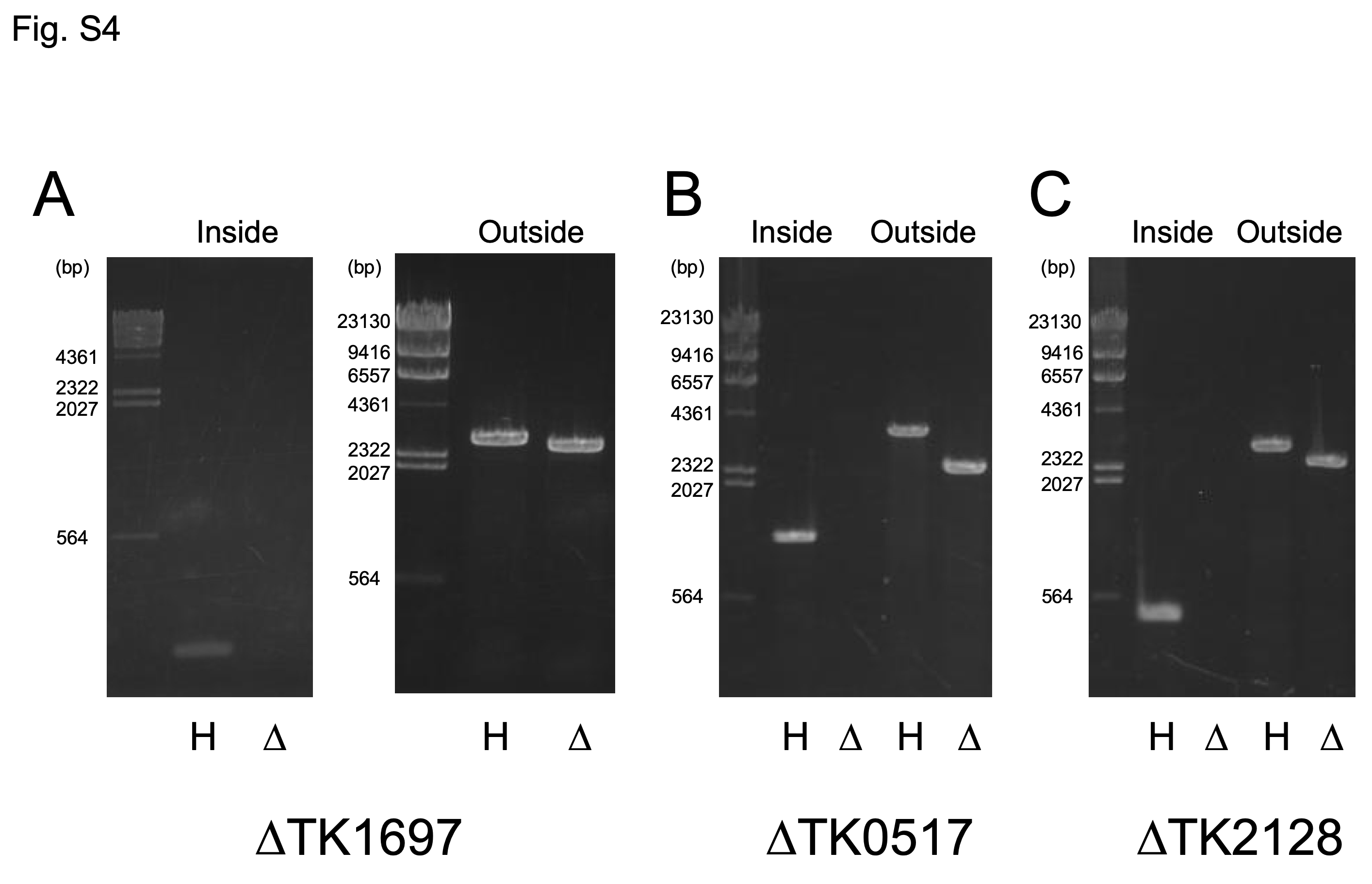

Supplement: FIG S4 [file mBio.01146-19-sf004.tif]

Fig. S5

A

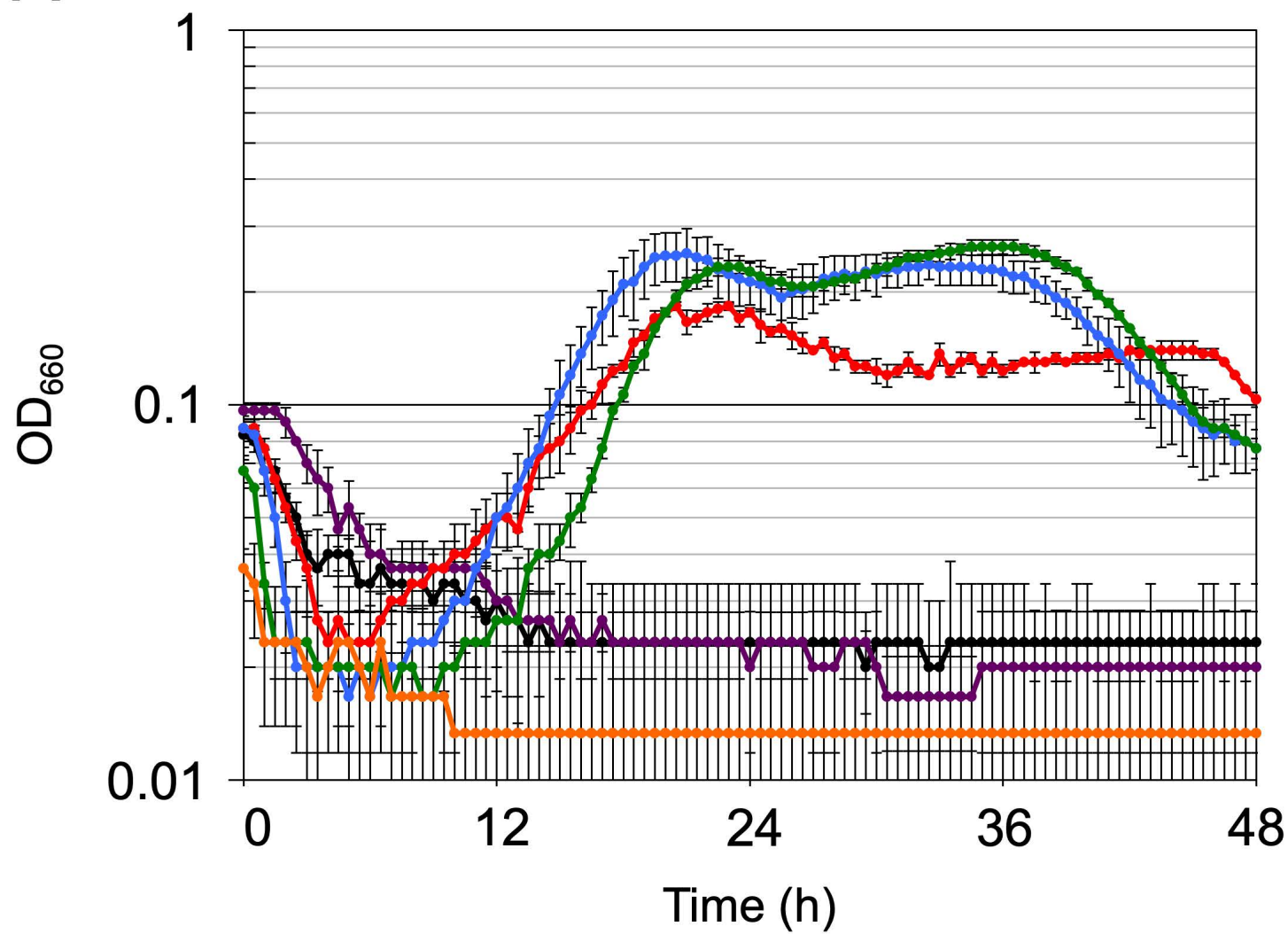

Fig. S5

B

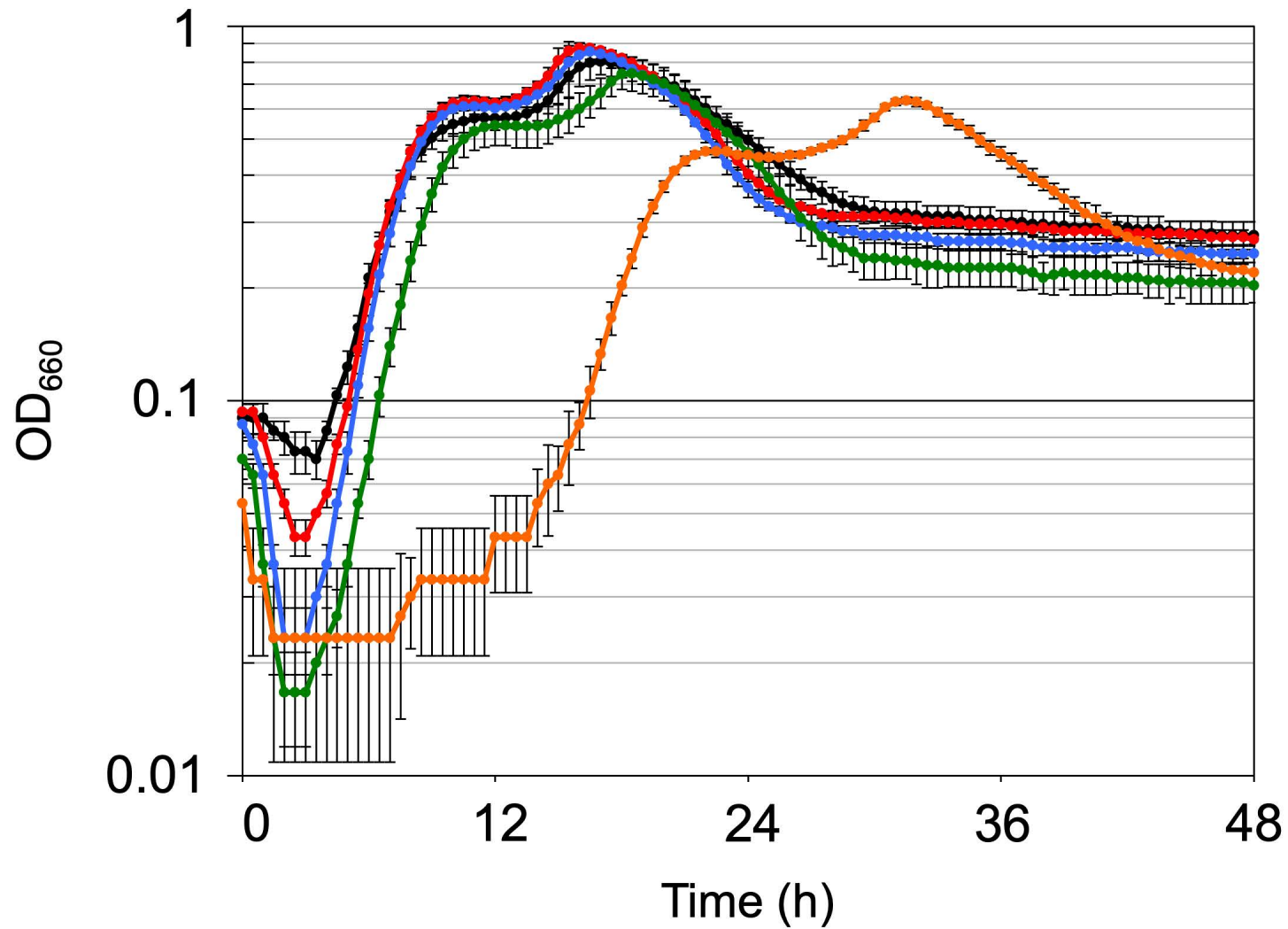

Supplement: FIG S5 [file mBio.01146-19-sf005.pdf]
