# Supplementary material for: Identification of Dephospho-Coenzyme A (Dephospho-CoA) Kinase in Thermococcus kodakarensis and Elucidation of the Entire CoA Biosynthesis Pathway in Archaea
Source: mBio. 2019 Jul 23;10(4):e01146-19. doi: 10.1128/mBio.01146-19 (PMC6650551; doi:10.1128/mBio.01146-19)
Supplement: FIG S6 [file mBio.01146-19-sf006.pdf]

Fig. S6

A

```
YP_919986.1    ------------------------------------MSSSP-----AARRELSYSWGYLFFDEPPLSVEKAWRLVQS-----SRFGKTL--ILVGDVVSK 52
YP_003816556.1 -----------------------------------------------MSGQLRR-------------IVEMQDWVKV--------------ICLGDVVSA 26
YP_007174639.1 --------------------------------MKFVGLTLP----ENVRYDFSQPRGRVISG-------ELKSYIES------REWNFV--ICVGDVVTY 49
NP_147085.1    ----------------------------MSVALSIPLLSLP----EDMREALAAAAGPVYSG------DRFRRQLLQ------TPCSGV--ACIGDYVSQ 54
YP_008603597.1 ----------------------------MTTKHLLPVLSLP----EDLRESLASAAGPVYSG------ERFVDEIAA------TPCGGV--ACIGDFVSQ 54
YP_006363113.1 --------------------------------MFLPSLVLR----EEFRRSASLPQGTLYIASKGGTVRGLEFT-----------------ATVGDVVSE 47
YP_001040844.1 ----------------------------MKSKFIVPVLKPE----SYLRKILSTPQGILYIG-----KYPIKDLEAH--------------ILVGDIVSN 49
YP_003669616.1 ----------------------------MKNKFIIPVLKPE----SYLRKILCSPQGTLYIG-----KYPIKDLEAH--------------ILVGDIVSN 49
YP_003650585.1 ---------------------------MASAGRSIPCLKPP----DHLRKALSLPQGKLYVS----DKQAITGLSCD--------------AAVGDVVSR 51
YP_004177111.1 ------------------------------MTRRLPVLKLP----EEHRSFLGIPQGDLYVSRTRGLVQGLEAD-----------------VAVGDVVSR 49
YP_002429036.1 ------------------------------MNKAIPVLKLP----VDFRLSLSIPQGDLYVSPDRGLVYGLRAD-----------------AAVGDIVSK 49
YP_006402879.1 ------------------------------MNKAIPVLKLP----VDFRLSLSIPQGDLYVSPDRGLVYGLRAE-----------------AAVGDIVSR 49
YP_004780378.1 --------------------------MHVASPPPGYCLEPS----EELREYMRRSKYLAYNT------AELSELSKR--------DDRL--AVIGDYTAR 54
YP_005841883.1 ---------------------------------------MN----DKLKKVLSEPFGPIYSG---SSFENLIKTTHN----------KFRYITIGDYVTE 44
YP_930015.1    ----------------------------------MTCFKLC-----CRRDLFAFPYPVAIWKEPPRSIEVVRDLVES------YGIEQI--YTVGDIVTT 53
YP_001794902.1 ----------------------------------MRCLRI-----RERRDLFAFPYPIAVWREPPRSLEVVSDLALS------YGVEHI--YTVGDVVTK 53
YP_001056647.1 ----------------------------------MTCYRLA-----KRRDLFAFPYPIAIWRDPPKSVEIVRNLVAD------GGFKHI--YTVGDVVTR 53
YP_001153914.1 ----------------------------------MTCYKLS-----RRRDLFAFPYPLTVWRDPPRSVEVVRDFVES------YDIRHI--YTVGDVVTR 53
YP_005259057.1 ----------------------------------MTCYKLS-----RRRDLFAFPYPLTVWRDPPRSVEVVRDFVES------YDIRHI--YTVGDVVTR 53
NP_560628.1    ---------------------------------------------MEVVKDIAESYG----------------------------AERI--YTVGDVVTQ 25
YP_005085004.1 ----------------------------------MICYRLA-----GRRDLFAFPYPIAIWRDPPSSVEFVKNLAES------YGAVHI--YTVGDVVTR 53
YP_004337001.1 -------------------------------------MKVP----EGRRHLFAFPYPVAIWRDPPRSVELAAEIARD------LGAARI--FTVGDVVTA 51
YP_004892291.1 ----------------------------------MRCLKLP----EERRELFAFPYPITIWRDPPRSVELALAVAGD------FGVKKI--YTVGDVVTA 54
YP_008796927.1 ---------------------------------MTNIWSRPIVFTEDMKNRVRKPLGELRHG-----DLNYSPELAQ--------AKPL--VVVGDYTFR 52
YP_003860570.1 ----------------------MLNDLGITKNGTAECFLMP----NELRNLLT--YFHIYKCSYVLTLRGDRESIGI-LLRLYYSNYVHETMVVGDYCCK 71
YP_008071435.1 -----------------------------------------------MRNELSTGFGELVKL------EDLKIQLEG--------CVKL--LSVGDFVSY 37
YP_007687666.1 ---------------------------------MSAGRRLQ----PSLREEFQKPVGRDIGI-------SELRTIDA---------KLI--IAVGDVVSL 45
YP_007713106.1 -------------------------------MGMAAGRRLT----PEMREVFQQPVGRDISE-------SELSQIHA--------KHII--ITVGDVVSL 48
YP_001541769.1 -----------------------------MSETNHPRLILR---SKRARALMALPFAISIPRDPPDSIMIARELFND------FKLSII--ATVGDVVSL 60
YP_003902647.1 -----------------------------------MYVLVS----KWARSMMAWPLGIAISKEAPDSIIRVRSEYGN---------SFL--ITVGDVVTM 50
YP_004244378.1 ------------------------------------MYVLA---SKWARSMMAWPLGIAISREAPESIVRVRSEYES---------SFL--ITVGDVVTM 50
YP_001737791.1 ---------------------------------MLFDLKLL----EDKRGKVSEIKGSIIKS---------LDILEN---------KRI--ISVGDRVTR 43
YP_023646.1    -----------------------------MQLKFDHDLVLN----QESINRIKEFSHSICS------IDDIKKHGGR-----------I--ITVGDVTTE 48
YP_008142358.1 -----------------------------MPLKLDRNIVIG----SRERKYIQDFKYSLCS------VDDIRELALK---------NKI--ISVGDVTTE 50
NP_111003.1    -----------------------------MLLRSGSKYIIT----AEAREEIKKRNGKLVT------IDEMVEVSKS---------TQL--DSVGDVTTT 50
NP_394550.1    ---------------------------------------MT----ESARQKIKGRNGRVVDP------DEVRDLSVD---------HPV--AAVGDVTAY 40
NP_341954.1    -------------------------MEVRNNNKVNLCFSF-----DNLRNELSRPYGILFIN-----NKIFLEFISK----SIQRGSRI--ITVGDYVSR 59
YP_005643228.1 -------------------------MEVRNNNKVNLCFSF-----DNLRNELSRPYGILFIN-----NKIFLEFISK----SIQRGSRI--ITVGDYVSR 59
YP_002915007.1 -------------------------MEIRDNNKVNLCFAF-----DNLRKELSRPYGILFTN-----NKVFLDFVSK----SIQQGFKV--ITVGDYVSR 59
YP_002829735.1 -------------------------MEIRDNNKVNLCFAF-----DNLRKELSRPYGILFTN-----NKVFLDFVSK----SIQQGFKV--ITVGDYVSR 59
YP_002843724.1 -------------------------MEIRDNNKVNLCFAF-----DNLRKELSRPYGILFTN-----NKVFLDFVSK----SIQQGFKV--ITVGDYVSR 59
YP_005648862.1 -------------------------MEIRDNNKVNLCFAF-----DNLRKELSRPYGILFTN-----NKVFLDFVSK----SIQQGFKV--ITVGDYVSR 59
YP_005646225.1 -------------------------MEIRDNNKVNLCFAF-----DNLRKELSRPYGILFTN-----NKVFLDFVSK----SIQQGFKV--ITVGDYVSR 59
YP_007866027.1 -------------------------MEIRDNNKVNLCFAF-----DNLRKELSRPYGILFTN-----NKVFLDFVSK----SIQQGFKV--ITVGDYVSR 59
YP_002832457.1 -------------------------MEIRDNNKVNLCFAF-----DNLRKELSRPYGILFTN-----NKLFLDFVSK----SIQQGFKV--ITVGDYVSR 59
YP_002840150.1 -------------------------MEIRDNNKVNLCFAF-----DNLRKELSRPYGILFTN-----NKLFLDFVSK----SIQQGFKV--ITVGDYVSR 59
YP_003419971.1 -------------------------MEIRDNNKVNLCFAF-----DNLRKELSRPYGILFTN-----NKLFLDFVSK----SIQQGFKV--ITVGDYVSR 59
YP_002837983.1 -------------------------MEIRDNNKVNLCFAF-----DNLRKELSRPYGILFTN-----NKLFLDFVSK----SIQQGFKV--ITVGDYVSR 59
YP_004457895.1 -------------------------MEIRDKYKVDLCFYLP----KEVRKELSRPYGILFKS-----TEKLLKYVDN--------FERI--ITVGDVVTQ 56
YP_001192301.1 -------------------------MEVRYKRKVDLCFRPP----RTVRAELSRPYGILFSN-----NAKLLSYLGQ--------FERI--ITVGDVVTS 56
YP_004408663.1 ---------------------------------------------------------------------------------------------------- 0
NP_376249.1    ---------------------------------------MP----DSIKKELTRPYGFLFTN-----NDIFINFILE------KKNNRL--ITVGDVVTS 44
YP_007433930.1 ---------------------------------------MP----RSLRAELSKPYGVLFIN-----ENKLKQFLIN------KSNSRL--ITVGDVVTQ 44
YP_007436201.1 ---------------------------------------MP----RSLRAELSKPYGVLFIN-----ENKLKQFLIN------KSNSRL--ITVGDVVTQ 44
YP_255496.1    ---------------------------------------MP----RSLRAELSKPYGVLFIN-----ENKLKQFLIN------KSNSRL--ITVGDVVTQ 44
YP_008946885.1 -------------------------MEIRNNSEIDLCFILP----RNLRAELSKPYGVLFIN-----ENKLKQFLKN------MSNSRL--ITVGDVVTQ 58
YP_001012648.1 ---------------------------------------------------------------------------------------------MGDRVSE 7
YP_006862428.1 -------------------------------------MPIN----ENDARLLKQPFGTLVPD-KQVTKRKVASVLKG--------VKQV--IAVGDATTE 48
YP_875902.1    -------------------------------------------------------MGTLILDSMVNKEAVLREAPPG---------TIL--VTVGDVTSE 34
YP_006776123.1 ---------------------------------------------------MKIPLGILIPE-NQTSKNEIQKYLSE--------NSYI--ITVGDRTTE 38
YP_001582567.1 ---------------------------------------------------MKIPLGILLSE-NQADKENILKHLEE--------NSYI--ITVGDRTTE 38
YP_006774212.1 -------------------------------------MKLP----DSLREQMKIPLGTLIPE-SQSNKENILKHIEQ--------NSYI--ITVGDRTTE 48
ZP_08257196.1  -------------------------------------MKLP----DSLRDKFKNPLGLLIPE-SQVNKSNIQKHLSK--------NPYL--ITVGDRTSE 48
ZP_08668423.1  -------------------------------------------------------MGILIPE-SQVSKSNVEKHLEK--------NSYI--ITVGDRTTE 34
YP_003400341.1 ------------------------------------MLKLT----EEVRAFAKKPFGLLYRGEG---VDVVKRVLKG--------DEFL--VCVGDIVSL 47
YP_003436628.1 ------------------------------------MLRMP----ESLRKDLQKPYGRVYRGRGVELVKRIEELRKA---------KYV--ACIGDLVSL 49
NP_069944.1    ----------------------------------MKGLRLP----ESMREELAKPHGKLYRGKGEKLLLEVEEISEA---------KPF--CTVGDLVTA 51
YP_007906114.1 --------------------------------MAVKGLKLP----DSLRDYLAKPHGRLYTGKGEDTVEKIEELGKY---------SPL--ICVGDLVTY 53
YP_001434673.1 -----------------------------------------------MRTLLASPRGALVKD------DRLLPFLLR--------GKTL--VTVGDVTTK 37
NP_614736.1    ---------------------------------MTVVLRLP----RELRPELRRPWGTLYPRPSIKTYRRLHEESEV-----------L--ITVGDMTTR 50
YP_003706636.1 ----------------------------------MNNYIMT----KELQSILKTPLGKIYKD-----IPEVFKIVKD----ISISNNLI--ISIGDITTK 51
YP_001323935.1 ------------------------------------MYILT----DKVATELKKPFGKVYKE---------LNSIEG----------KV--ISIGDVTTK 39
YP_001330653.1 ------------------------------------MYLLT----DEVARELKKPFGKVYKE---------LPSIDG----------KV--VSIGDVTTK 39
YP_001097707.1 ------------------------------------MYLLN----DKVAHELKKPFGKVYKE---------LPSIEG----------KV--VSIGDVTTK 39
YP_004742083.1 ------------------------------------MYLLN----DKVAHELKKPFGKVYKE---------LPSIEG----------KV--VSIGDVTTK 39
NP_987562.1    ------------------------------------MYLLN----DKVAHELKKPFGKVYKE---------LPSIEG----------KV--VSIGDVTTK 39
YP_001548519.1 ------------------------------------MYLLN----DKVAHELKKPFGKVYKE---------LPSIEG----------KV--VSIGDVTTK 39
YP_001324483.1 ------------------------------------MYMLN----ESTKEILKKPFGKVYKE---------LPPINR----------KVNIIAVGDITTK 41
YP_004484635.1 ------------------------------------MYILT----DELRKLLKKPFGKVYKE---------LPPING----------KV--VSVGDITTK 39
YP_004575995.1 ------------------------------------MYILN----ERLREILKKPFGKVYKE---------LPLING----------KV--ISIGDITTK 39
YP_003616685.1 ------------------------------------MLKLP----DHLREELRKPFGKVYKE---------FPDVDG----------YI--VTVGDIVTK 39
YP_003247808.1 ------------------------------------MLILP----EELRDLLKKPFGKVYKT---------LPHIDG----------DI--ITVGDIVTK 39
YP_003128192.1 ------------------------------------MLKLP----EDLRETLKKPFGKVYKT---------LPNIDG----------DI--VTVGDIVTK 39
NP_247369.1    ------------------------------------MLVLP----EELREKLKKPFGKVYKT---------LPDIDG----------DI--VTVGDIVTK 39
YP_003458341.1 ------------------------------------MLMLP----EELREKLKKPFGKVYKT---------LPDIDG----------DI--VTVGDIVTK 39
YP_008075056.1 ------------------------------------MLQIT----TKLIQELKDPLGELYPN-----FEDALEDIKA--------SKFL--ISAGDCTLN 45
YP_001272768.1 ------------------------------------MLRLDAELNKDTISKLKKPLGKLYPH-----FEDAIEEIKS--------SEFL--ISVGDATFN 49
YP_003424221.1 ------------------------------------MFRID----ENSIDEFKKPLGVLYPD-----FEDAIPMIKE--------ASFL--ISVGDQTIK 45
YP_004004002.1 ------------------------------------MLILP----KKLRKKLKKPFGKLYKS-----IDDIKNFPPN--------EYKI--ISVGDETTK 45
NP_275409.1    ------------------------------------MYILP----EELRAELKRPLGELHRS---------FSDVDV-------GNSFL--ITVGDVTTR 42
YP_003849624.1 ------------------------------------MYILP----EKLRAELKKPLGELHES---------FRDIDV-------GDSFL--ITVGDVTTR 42
YP_447663.1    ------------------------------------MLTLP----KYLRSELKKPLGQLYKS-IDIIEEKLHQQLSE--------DKLI--ISIGDATTK 49
```

Fig. S6

```
YP_004289823.1 ------------------------------------MLILK----QDMRSEFKKPIGTLFPT-----ISDAKDSLDK-----KGEKGLL--ISIGDVTTR 48
YP_004520574.1 ------------------------------------MLVLK----KELRSEFKKPFGKIYPS-IADTEEPLKESLGD--------DGVI--ISIGDVTTQ 49
YP_008916228.1 ------------------------------------MLILK----QELRSEFKKPLGVLYSS-----LDEAEDFIKS-----KYPDELL--ISVGDITTQ 48
YP_003482939.1 --------------------------MENTHYELDKPLLLK----EETRKKLQKIYGKLISE-------DELKNIEG----------EI--FSVGDVVTH 51
YP_007246537.1 --------------------------MESMPYELDKPLILP----EHLRGELQRVHGILLEE-------DEIKNIEG----------MI--ICVGDVVTH 51
YP_004341579.1 ------------------------------------MLRLP----EELRKECRKPQGRLCS----------LDSVNP-----------V--AAVGDVVSY 37
YP_843741.1    ----------------------------------MRVLVLP----EELRDELKEPLGVLYRCHGVECVERMSDHLRA--------AERV--IAVGDITTF 52
YP_004385292.1 --------------------------------------------------MLKSPLGKLCKGNGTECVEAMRDELQG--------AKKV--AAIGDMTAF 40
YP_005920892.1 ----------------------------------MKILRLP----EELRSELKPPLGTLFRGRGQECIGPMQDLLRP--------APKV--IAVGDVTTF 52
YP_685355.1    -----------------------------------MIYKLT----PALRDDLKTPFGTLYPGKGESCLAKVVKALDK--------PPKI--ISIGDVTTF 51
YP_003357728.1 -----------------------------------MAYRLT----AALRDRLKTPFGRLYRCKDDACLRGVVESYKG--------APKV--IAIGDVTAY 51
YP_005379469.1 -----------------------------------MAYRLT----PALRERLKAPFGELYRCKGEECLRGILKGFKS--------PPKV--ISIGDVTTY 51
YP_303847.1    ---------------------------------MSVHIELP----RELRPLMKKPLGTLYRGKGRDTIEKFAGELGS--------PTKL--ISVGDVTTF 53
NP_618569.1    ---------------------------------MSVHIELP----TELRPLMKRPLGTLYRGKGRDTVEKFVGELAS--------PTKL--ISVGDVTTF 53
NP_632622.1    ---------------------------------MSVHIELP----RELRPLMKKPLGILYRGKGRDTIEKFAGELSS--------PTKL--ISVGDVTTF 53
YP_007489295.1 ---------------------------------MSVHIELP----RELRPLMKKPLGILYRGKGRDTIEKFAGELSS--------PTKL--ISVGDVTTF 53
YP_003726277.1 ---------------------------------MGSQIILP----EKFRPTLRKYFGELFLGTGNDTIKKMEQQLKN--------STKL--ISVGDVTTF 53
YP_003541665.1 ---------------------------------MGGRISIP----ASLRPLLRKKFGVLYKGSGEETVGKVSGDLGD--------PIKL--ISVGDVTTF 53
YP_004615575.1 ---------------------------------MGEKIILP----PEFRPLLRVPFGTLFTGDGRDTIQKVLECMGN--------PTKL--ISVGDVTTF 53
YP_566892.1    ---------------------------------------MP----DSLRPLLRKPFGVLYTGIGSDAVKSLVKDLNN--------PTKL--ISVGDVTTF 47
YP_006922550.1 ---------------------------------------MP----ESVRPLFRKLFGTLYTGTGDDTIRKLSKDLDS--------PTKL--ISVGDVTTF 47
YP_007312351.1 ---------------------------------MVETIHLP----EELRPLLRKTFGVLYTGTGDDTIQKLSKDLGS--------PTKL--ISVGDVTTF 53
YP_657114.1    --------------------------------MDRLPLELP----SSLRQEFKSPFGPVYTD-----VTQFLTDAGR----------PI--IAVGDIVTY 47
YP_005839125.1 --------------------------------MARLPLELP----SSLRQEFKSPFGPVYTD-----VTQFLADAGR----------PI--IAVGDIVTY 47
YP_008055518.1 ----------------MSGGEDSGTSADRSASEPDVVLELP----EAARGEFKDPFGPVYTD-----VDALLAEHSG----------PI--VTVGDIVTY 63
YP_001689848.1 ------------------------------MSETDAAATLP----ADARDAFKSPIGPVYTD-----AAALLADATP----------PI--IAVGDVVTY 49
YP_003735962.1 ------------------------------MADPEVLLRLP----DSLRGAFKEPMGPIETD-----AAVLLEGVSG----------PL--IAVGDVVSS 49
YP_002567039.1 ------------------------------------MLTLP----ESLRDAFKEPLGPVTID-----ADELLAAAAETRGERAAPDAPI--IAVGDVVTY 53
YP_007285258.1 -------------------MTDSSPSDADDASPPEPLVSLP----KSLRHELKDPIGPVETD-----PHSLLEVAGE----------PL--VTVGDVVTY 60
YP_004808741.1 ------------------------------------MLTLP----PELRDELKAPFGPLYTD-----TETLLSESGR----------PI--IAVGDVVTY 43
YP_004035542.1 ------------------------------------MLRLP----TALRGEFKEPFGPLYTD-----ADRLLADAGT----------PI--IAVGDVVTH 43
YP_003535927.1 ------------------------------------MLTLP----TALRAAFKEPFGPVYAE-----ADDLLADASS-----EDADAPL--VAVGDVVTF 48
YP_006349668.1 ------------------------------------MLTLP----TDLRAAFKEPFGPVYTE-----AEALLADASS-----EETDAPL--VAVGDVVTF 48
YP_007486518.1 --------------------------------MSTILAKLP----TGMRAELKEPLGAVYTD-----ADALLADAGE----------PL--VAVGDIVTY 47
YP_331261.1    --------------------------------MSKILAKLP----TEMRGELKDPLGEIYTD-----TDELLADAGD----------PL--VAVGDIVTY 47
YP_003479628.1 ---------------MTPDDTSTNAAGRDRNQEYEQLLVLP----DDLRHELKEPMGPIETD-----AEQLLESVDG----------PL--IAVGDVVTY 64
YP_007309925.1 ----------------------MTRDDSDPDRADEQLLVLP----DELRHELKEPMGPIETD-----ADVLLEDVSG----------PL--IAVGDVVTY 57
YP_003404991.1 MTRDDSPAETDTDADAAANANADTNGAASTPDIDDQLLVLP----DDLRHELKEPMGPVETD-----ADRLLKDVDG----------PL--IAVGDVVTY 79
YP_006541826.1 ---------------------MTRDDEAPTLTADEQLLVLP----DDLRHELKEPMGPIETD-----AERLLETVDG----------PV--IAIGDVVTY 58
YP_007279381.1 ---------------------MTRDDTDDAFADDDQLLVLP----DALRHELKEPMGPIETD-----ADRLLEGVDG----------PL--IAVGDVVTY 58
YP_004597979.1 ------MTDDTDSDADAIEDVEDAETAETVETDYEHLLTLP----DGLRAELKEPLGPIETD-----AERLLADVDG----------AL--IAVGDVVTY 73
YP_007177942.1 ---------------MTGDEYDSDPTDGAVSDPADQLLVLP----EELRSELKEPMGPIETD-----VESLLEAAGE----------PL--IAVGDVVTY 64
YP_003131105.1 ------------------------------------MLSLP----ESLRSALKEPFGPVRTD-----ADSLLAEAGD----------PL--IAVGDIVTY 43
YP_008377469.1 ----------------MSEEPSTPLDRDGSGAGGDVVLSLP----ESLRSALKEPFGPVFTD-----ADSLLAEAGD----------PL--IAVGDIVTY 63
YP_003176564.1 --------------------------------MSSLRLDLP----SSLRHELKEPLGPIYTD-----ADALLADATE----------PV--VAVGDIVTY 47
YP_004794731.1 --------------------------------MSDVVLELP----SDLRHELKEPLGRIYTD-----TAALLADAGD----------PI--IAVGDMVTY 47
YP_008874633.1 --------------------------------MSDVVLELP----SDLRHELKEPLGRIYTD-----TAALLADAGD----------PI--IAVGDMVTY 47
YP_504276.1    ------------------------------------MRILP----ARHRQLFKEPFGTLFHS-----FEEVLKFLPG---------KTV--FSVGDVVTA 44
YP_001030096.1 ------------------------------------MLTLP----PENRWLFREPFGTVFSD-----FSMVLPYIEG---------KIF--CTVGDVVTH 44
YP_003893312.1 -----------------------------------MNWYLP----ERHRDRFKEPFGRLFPD-----IRAALEYSGG---------APV--FAVGDVVTY 45
YP_001403408.1 ------------------------------------MFVLP----DESRQLFKDPFGTLHRD-----IGTVLPELAG---------RTI--YSVGDVVTH 44
YP_007250359.1 ------------------------------------MLTLP----EEHRKLFKDPFGELYPE-----ITDILPLLSQ---------KTV--FTVGDVVTY 44
YP_002465695.1 ------------------------------------MLRLP----DEHRALFRNPFGTLYPE-----FSMVLPHLAG---------RAI--YTVGDVVTA 44
YP_001048149.1 ------------------------------------MLRLP----EAHRDLFKKPFGTLYTS-----VDELLPRLEG---------RAV--YAVGDVVTH 44
YP_006545055.1 ------------------------------------MLRLP----EAHRDLLKIPFGTLYRD-----IGELLPLIEG---------RAI--YAVGDVVTH 44
YP_002582924.1 -------------------------------MCDDFYFYLP----PALRDELKEPLGELVRGEIPEPYLRVLPLLRE--------APFL--ITVGDVVTE 55
YP_002958867.1 -------------------------------MCGDFYFYLP----PSLRDKLKEPLGELVRGEIPEPYLRILPLLRK--------VPFL--ITVGDVVTE 55
YP_002308299.1 ----------------------------------MFHFKLT----PELRKELKDPLGKLIRGEIPKPYLMIREELER--------ARHV--VTVGDVVTE 52
YP_004762704.1 -----------------------------------MRFVLT----PELRRELKDPLGELIRGELPEPYIRLKGELER--------AGHV--VTVGDVVTE 51
YP_006424759.1 -----------------------------------MRLVLT----PELRKALKEPLGELVRGEIPEPYVKIREELEK--------ARHV--VTVGDVVTE 51
YP_004072211.1 -------------------------------MCREFYFKLP----FSLRSELKKPLGELVRGELPRPYILVKEKLEK--------APYL--ITVGDVVTE 55
YP_002993448.1 -------------------------------MSRNFYYKLT----EDLRNELKKPLGELIEGKMPQPYLKSREELKK--------APFL--VTVGDVVTE 55
YP_008428988.1 -------------------------------MSREFYFKLT----EDLRKELKNPIGRLIKGEMPQPYLISRRELEN--------APYL--VTVGDVVTE 55
YP_004624278.1 ---------------------------------MTVLFRLP----PELRGELKRPLGELIRGPIPGPYLKVRERLAR--------ARNL--VTVGDVVTE 53
YP_006354082.1 ---------------------------------MRVIFKLP----LSLRHELKKPMGKLIRGEIPRPYLEIRDKVSN----------PL--ITVGDVVTE 51
YP_004423817.1 ---------------------------------MRVLFKLP----EKLRRELKKPIGQLIPGKMPEPYLKVREIVER--------ESLL--ITVGDVVTE 53
NP_577983.1    ---------------------------------MSVMFKLP----LNLRQELKKPLGELIKGEIPIPYLKLKEIVNS---------NTL--ITVGDVVTE 52
YP_006491333.1 ---------------------------------MSVMFKLP----LNLRQELKKPLGELIKGEIPIPYLKLKEIVNS---------NTL--ITVGDVVTE 52
NP_127361.1    ---------------------------------MKVLFKLP----PSLRSELKKPVGELIEGDIPTPYLKVKDILTN--------EDPL--VTVGDVVTE 53
NP_143738.1    ---------------------------------MRVVFKLP----DELRQELKNPLGELIEGNIPEPYVKAKNIIEG-------DDGVL--ITVGDVVTE 54
Jpred 4 SS     -----------------------------------EEEE------HHHHHHHH----EEE------HHHHHHH--------------EE--EEEEEHHHH
TK1697         ---------------------------------MKMFFRLT----RELRDELKRPLGELVRGPIPEPYLKVRGELEK---------HPV--VTVGDVVTE
CONSENSUS 0.95 ---------------------------------------------------------------------------------------------GD----

YP_919986.1    SF---RERG--LGD---FFIVDGHTRRNLK--VEDL-PGK---AE---------AF--TCKNRPGEISRECYELLR--NLLTSAIGRGKKIVVYVEGEED 125
YP_003816556.1 YC---MESA-RLPD---VIVFDGKTQRAESKFVKED-LLR---GM------GFGLV--EVSNPPGGLSVEAMETLC--DAIN---SYNGRIAIKVNGEED 102
YP_007174639.1 YC---LKAN-RKPD---IIIIDGKTLRSKE--GFSF-DSN---PI------DYNLI--KIKNPPGMLTYDNIDLLC--NI--LKSKNKEKILIYVDGEED 124
NP_147085.1    ACIATLSTAWTGPL---ILVVDGKTRRESW--RDMV-VPQ---GF--------RVH--RVRSPPGSLSLEAYTTIC--KLM----EEYGRHVVFVEGEED 129
YP_008603597.1 IC---LSALSRRPDTPLLLVLDGKTRRESG--VATG-VPE---GF--------KLY--QARSPPGHLSLEVYSLVC--SAI----EGGGRSAVVVEGEED 129
YP_006363113.1 T---------LESS---IKVVDGKTRRGDH--RSSV-VGV---AS--------------IVNPRGAVSLHSLTVLR---------GCRSGTYVVI-GEED 105
YP_001040844.1 T---------LRGN---IKIIDYKTRRHIN--IKPI-INE-------------PLT--NLVNPRGTMSL-MSRTIA---------KAKNYRTIIVKGEED 109
YP_003669616.1 TL----RGN--------IRIIDYKTRRRIN--IKSI-IKK-------------PLA--NLVNPRGTMSL-MSRTIA---------KAKKYRTIIVKGEED 109
YP_003650585.1 N---------HYAR---IRVVDYKTKRNIP--TGKG-PAG---SR---------VF--RVVNPPGCVSLNSF-TIG---------FLKGEHVVEVIGEED 112
YP_004177111.1 NH--------TVA----LRVTDAKTKRSMT--VDTG-LSR---CD---------TT---IVNPPGSISMQAFTASL---------MKQAKGICVI-GEED 109
YP_002429036.1 NH--------MVE----MRITDAKTKR-HT--VDVG-PGE---CD----------V--LVVNPQGTISINSFTSS----------LIGGARSICVVGEED 108
YP_006402879.1 NH--------MVE----MRITDAKTKR-HT--VDVG-PRE---CD----------V--LVVNPQGTISINSFTSS----------LMGGARSICVVGEED 108
YP_004780378.1 LM---LKHG-LHPH---IVIIDCLTRR-ER--IECI-NAP---PG-------YRVV--RAWNPRGYISWDSWSVIA--EAW----ERSNPTMILIEGEED 125
YP_005841883.1 KY---IEIAEKSPV---LSFVDMQTKRERYDISKIK-SYY---TD---------II--EIYNKQGTISKETINEEL--SEVLINYIQGISSLVIVRGEED 121
YP_930015.1    NF---LKYS-LAPT---SAAVDGKTRRGLK--IDKP-TFF---RK---------TI--EVYNPPGYITEEAWIAVE--EAV------RDNVMIKVNGEED 121
YP_001794902.1 NF---LDYG-VTPT---SAAVDEKTRRGLP--VEQH-RKF---QR---------VI--KVVNPPGYITEEAWAAVE--EAV------GGGVLIKVEGEED 121
YP_001056647.1 NF---LEYG-LIPT---SAAVDEKTRRGIR--VERF-SAF---RS---------VV--EVVNPPGYITDEAWSAVE--KAV------EGGVVVKVRGEED 121
YP_001153914.1 NF---LEYG-LAPT---SVAVDEKTRRGIK--VEGL-GLY---KR---------VI--RVNNPPGYITEEAWAAVE--EAV------EGGVLIKVDGEED 121
YP_005259057.1 NF---LEYG-LAPT---SVAVDEKTRRGIK--VEGL-GLY---KR---------VI--RVNNPPGYITEEAWAAVE--EAV------EGGVLIKVNGEED 121
NP_560628.1    NF---FKHG-LIPT---SAAVDEKTRRGVR--IEQL-AVF---KR---------VI--KVNNPPGYITEEAWSAVE--EAV------RGGVIIKVEGEED 93
YP_005085004.1 NF---LSQG-LVPT---SVAIDEKTRRGVK--VEQL-NLF---KR---------VI--RVVNPPGYITEEAWAAVE--EAV------GGGVVIKVEGEED 121
```

Fig. S6

```
YP_004337001.1 NF---LAAG-LAPD---AAAVDLKTRRSEY--VRTV-EAF---PR---------IL--RVRNPPGYITEEAWAAVE--YA-----VKTPGTLLLVDGEED 120
YP_004892291.1 NF---LAAG-VVPN---AAAIDLKTRRREY--VEIT-TRF---PH---------VL--KVRNSPGYITEEAWRAVQ--TA-----VNNDGTLLLVEGEED 123
YP_008796927.1 RM---IEAG-LRPD---VVVVDAKVMRNAA--EMPR-LEG---YE---------VT--RVRNMAGVIEPEAAEAVM--SA-----VKKGRGAVFVDGEED 121
YP_003860570.1 TY---IEFV-GVPR---LCIVDGKTLRYNN--TDIR-DIE---RR------FNYVE--RCVNPRGCITTNCMEAIK--RCY----KEGVPCLLIVDGEED 144
YP_008071435.1 TL---LDNG-FKPD---LIIYDLKTER-RP--FSPL-SSK---LS----LLCGEEV--VVKNPAGYITPELVTEIS--SAL----GRDAPTKIRVDGEED 111
YP_007687666.1 TM---RENG-IVPL---LSVYDGYTERREL--TGFADLVR---NT------GLEET--VVSNPAGTVTSQLEDAVE--NAL----AGRSAGIIRVEGEED 119
YP_007713106.1 TV---RKHG-IVPL---LSVYDGYTER-RE--LTGF-ADL---VR----DAGLAET--VVSNPAGTITSQLSDAVE--NAL----AGRSAGIIRVEGEED 122
YP_001541769.1 NV---ATYW-RRPD---LRVIDLNTRRGTV--IQHS-DMD---GV---------VY--RVRNERSTLSYESFNIMR--SAYANV-LSGNRVTIIVDGEED 133
YP_003902647.1 NT---IRYW-RVPD---LAFMDLKTRRTIG--MGGV-ETV---GF-------DRVI--NIVNKPATLSIEVINAVR--DAMRDA-VNGIHVLIQVDGEED 125
YP_004244378.1 NT---IKYW-RVPN---LAFMDLKTRRTID--MGME-ITG---FD--------KVI--NIINRPATLSIEVINAIR--GAMREA-MGGSRVLIQVDGEED 124
YP_001737791.1 EL---LGSG-RRPE---VAIIDLKERREMN--CSTI-FYL---DD-------YLIL--VARNPAGTLMREAWLKVR--KAIEIS-LSGKNAAVIVDGEED 118
YP_023646.1    NL---ERAG-LDIF---IEVVDLKTKRGEK--TYNH-REN---SI-------------SIENRPGTISLSLIRAIE--SSI----KSNKKTRIEINGEED 116
YP_008142358.1 NL---KNAG-IKLN---LQIVDLVTKR-DG--KRFM-HEE---GS----------I--TVSNPPGIISIDLMNKIY--EFI----STGVHGRIEVDGEED 118
NP_111003.1    NL---RRAG-AKLF---LQIVDLKTKRGES--IFQH-EEG---SI-------------TVRNDPGTISFDLIEAIK--SSI----ENNKPTRIEVIGEED 118
NP_394550.1    NL---RKVN-ARVL---LEIVDLRTKRSDL--LFAH-EEG---SI-------------VIRNDPGTISFDLIRAIQ--SAL----LSDKKTRIEVIGEED 108
NP_341954.1    VL---EENG-IVPF---LEVIDGKTKRTIT--QNRA-ITR---NK---------EY--KVTNEAGKIRFEIFEIME--NIL----KDREGGVVFVDGEED 129
YP_005643228.1 VL---EENG-IVPF---LEVIDGKTKRTIT--QNRA-ITR---NK---------EY--KVTNEAGKIRFEIFEIME--NIL----KDREGGVVFVDGEED 129
YP_002915007.1 VL---EENG-IIPF---LEVIDGKTKRSIP--QHRA-IVK---NK---------EY--RVTNEAGKIQFEIFEIIE--NIL----KDRDGGVVFVNGEED 129
YP_002829735.1 VL---EENG-IIPF---LEVIDGKTKRSIP--QHRA-IVK---NK---------EY--RVTNEAGKIRFEIFEIIE--NIL----KDRDGGVVFVNGEED 129
YP_002843724.1 VL---EENG-IIPF---LEVIDGKTKRSIP--QHRA-IVK---NK---------EY--RVTNEAGKIRFEIFEIIE--NIL----KDRDGGVVFVNGEED 129
YP_005648862.1 VL---EENG-IIPF---LEVIDGKTKRSIP--QHRA-IVK---NK---------EY--RVTNEAGKIRFEIFEIME--NIL----KDRDGGVVFVNGEED 129
YP_005646225.1 VL---EENG-IIPF---LEVIDGKTKRSIP--QHRA-IVK---NK---------EY--RVTNEAGKIRFEIFEIME--NIL----KDRDGGVVFVNGEED 129
YP_007866027.1 VL---EENG-IIPF---LEVIDGKTKRSIP--QHRA-IVK---NK---------EY--RVTNEAGKIRFEIFEIME--NIL----KDRDGGVVFVNGEED 129
YP_002832457.1 VL---EENG-IIPF---LEVIDGKTKRSIP--QRTI-VKN---KE----------Y--RVTNEAGKIRFEIFEIME--NIL----KDRDGGVVFVNGEED 128
YP_002840150.1 VL---EENG-IIPF---LEVIDGKTKRSIP--QRTI-VKN---KE----------Y--RVTNEAGKIRFEIFEIME--NIL----KDRDGGVVFVNGEED 128
YP_003419971.1 VL---EENG-IIPF---LEVIDGKTKRSIP--QRTI-VKN---KE----------Y--RVTNEAGKIRFEIFEIME--NIL----KDRDGGVVFVNGEED 128
YP_002837983.1 VL---EENG-IIPF---LEVIDGKTKRSIP--QRTI-VKN---KE----------Y--KVTNEAGKIRFEIFEIME--NIL----KDRDGGVVFVNGEED 128
YP_004457895.1 EL---LKNN-RRVF---LSIVDGKTKRNIK--LHMR-FSN--------------YI--MVKNEPAIIRLSAMSYIK--AAL----NSSFSSVIYVDGEED 124
YP_001192301.1 LV---TRSG-IRPF---LSVVDGKTRRNVS--ISGERSSE------------------VITNEAGILRFSAMSKIK--EIM----YGREPRSLFVNGEDD 123
YP_004408663.1 ----------MTPF---LSIIDGKTKRNKT--LDHR-ETE---------------F--RVINEPGILRLSVMSKIK--SIM----ENSKPTSLFVDGEDD 61
NP_376249.1    TL---YDRN-IIPF---LSIIDGKTKRQIK--VKTV-NEN--------------VI--KVKNEKSTIRFSVMKIIK--NLL----DKNERATILVDGEED 112
YP_007433930.1 TI---LKFH-IKPF---LAIVDGKTKRKLV--K-EN-FGQ---FE---------YI--KVKNEPGLIRLSTIREIK--DILV---HNENDKILMVDGEED 114
YP_007436201.1 TI---LKFH-IKPF---LAIVDGKTKR-KL--VKEN-FGQ---FE---------YI--KVKNEPGLIRLSTIREIK--DILV---HNENDKILMVDGEED 114
YP_255496.1    TI---LKFH-IKPF---LAIVDGKTKR-KL--VKEN-FGQ---FE---------YI--KVKNEPGLIRLSTIREIK--DILV---HNENDKILMVDGEED 114
YP_008946885.1 TI---LKFN-IRPF---LAIVDGKTKR-KL--VQEN-FGQ---FE---------YL--KVKNEPGLIRLSTIRVIK--DI--LV-YNEKDRILMVDGEED 128
YP_001012648.1 TL---IRHG-VKPW---VMVFDCVEARRDK--TCPS-IPE---GY--------AIL--RTRNERSTVEPGAVEVIR--KA-----LRQGHTVVRVDGEED 77
YP_006862428.1 RL---VSFG-IKPD---IAVIDGKERRSKR--SYSA-SYS---AK---------EL--HCINPAGTISKEAVQVLQ--DAL----RMPSPVRVLVDGEED 118
YP_875902.1    RI---SGFG-MTPL---LQIIDGKTRRAAH--EPAGPPPD---VE---------II--RCENPAGGISPECIETIR--RAL----GSSSPLRLVVSGEED 105
YP_006776123.1 KM---IDFD-LIPS---LQIIDGQEKREKR--IPPK-LKN---AI---------EL--TVDNPPAEITSQSISVIK--KAF----TMKSPVRIFVNGEED 108
YP_001582567.1 KM---IDFD-LIPS---LQIVDGIEKREKR--EPPK-LVN---TT---------EI--TVDNPPAEITSQSIDVIK--KAF----SMESPVRILVTGEED 108
YP_006774212.1 KM---IGFD-LIPS---LQIVDGIEKREKR--EPPK-LAN---AT---------EI--TVDNPPAEITSQSIDVIK--KAF----LMKTPIRILVTGEED 118
ZP_08257196.1  KM---VDFG-FIPS---LQIIDNQEKRVKR--EPTKNIHG---YT---------EL--VCENPAAEITPQSVEIIK--KAF----ESKPPVRLTIIGEED 119
ZP_08668423.1  KM---ISFG-LTPS---LQIIDNYEKR-VK--REPT-MSP---TV-------YTEL--TCNNPAAEITPASIDIIK--KAF----ASKTPVRLTVVGEED 105
YP_003400341.1 TT---LKAG-FKPK---IIVFDGKSIRREL--NSVV-------NRIRDLSRGYKEI--VAENPAGCITEDLAEKVF--EAVNSA-LEGLNVRIYVRGEED 126
YP_003436628.1 YS---LQAG-YKPD---IVVLDYKTEREKL--DDDFSSKI---SK---LLEGYEKV--EAENPQSCISEDLVEKLV--EAVRN--LGRRRTAVIVKGEED 128
NP_069944.1    SA---IKVG-LTPD---LAVADGKTLREEN--VEFEQEAF---DE---------VI--ETTNPPAHISCELISALL--KALKLC-EDGKKVLVFVDGEED 125
YP_007906114.1 YI---FKAG-YNPD---IVVIDFKTIRNEI--DEEV-ISFIESKT-----EGYVRL--DVVNPQAYITLDLVEKLI--EAVS---LLPKKVLIVVEGEED 131
YP_001434673.1 RC---LELG-LVPR---TAIFDGKTRRSQW--VELR-APN---GV----------L--KAFNPPGQICLDAAKVVK--KAILTS------TWVKVEGEED 104
NP_614736.1    SF---LRCS-IRPD---VAVVDRKMLRTVP--VDPG-NKF---PV---------TL--DVNNPPGTITREAWDTVR--RGIDYA-LDGDATLIDVTGEED 123
YP_003706636.1 NL---IEYG-IIPK---ISILDFKTKRDIP--VDIS-YNF---KK---------VY--TVDNPAGTISQTSMEIIK-----YLSDIDDREVALIVAGEED 122
YP_001323935.1 HL---LSNG-IVPD---LSILDFKTKRNVP--VEIP-HKF---KT---------VF--EVYNPPGYISDEAIERIK-----YLSTIDDRDMALIVKGEED 110
YP_001330653.1 HL---LSNG-IIPN---LSILDFKTKRNIP--VNIP-HKF---KT---------IF--EVENPQGCISDEAIERIK-----YLSTIHDRDMALIIKGEED 110
YP_001097707.1 HL---LSNG-IIPN---LSILDFKTKRNIP--VNIP-HKF---KT---------IF--EVENPQGCISDEAIERIK-----YLSTIHDRDMALIIKGEED 110
YP_004742083.1 HL---LSNG-IIPD---LSILDFKTKRNIP--VDIP-HKF---KT---------IF--EVENPQGCISDEAIERIK-----YLSTIHDRDMALIIKGEED 110
NP_987562.1    HL---LSNG-IIPD---LSILDFKTKRNIP--VDIP-HKF---KT---------IF--EVENPQGCISDEAIERIK-----YLSTIHDRDMALIIKGEED 110
YP_001548519.1 HL---LSNG-IIPD---LSILDFKTKRNIP--VDIP-HKF---KT---------IF--EVENPQGCISDEAIERIK-----YLSTIHDRDMALIIKGEED 110
YP_001324483.1 NL---LSKS-IIPN---LSIIDLKTKRNIP--VKIN-HKF---KT---------VF--EVNNPQGCISDEAEETIKYISSL----DMVDTALIVKDGEED 113
YP_004484635.1 TL---LSKG-IIPN---LSIFDLKTKRDIK--VDIE-HKF---KK---------TF--EVENPPGCISDEAIEKIK-----YLANINDKDIALLVKGEED 110
YP_004575995.1 NL---LSKN-IIPN---LSIFDLKTKRDIS--VEIN-HKF---KK---------VF--EINNPPGCISDEAMEKIK-----YLSTRNNEDVALKVNGEED 110
YP_003616685.1 SA---IEKG-VKIK---LAIYDLKTKRNIP--VKIN-YKF---KK---------TF--KVCNPPGYISDEAIEKIK-----YISQLNDDDIGLYVEGEED 110
YP_003247808.1 TV---IENG-IIPK---LSIFDLKTRRNIP--IKIN-HEF---KK---------VI--KIKNPAGCISNEAIESIK-----YLSTINDKNNAILVDGEED 110
YP_003128192.1 TV---IENN-ITPK---LSIFDLKTQRNIP--VKIN-HVF---KK---------II--KVKNPPGCISDEAIESIK-----YLSTINDKDIALLVDGEED 110
NP_247369.1    TA---IENN-IIPK---LSIFDLKTRRNIP--VKIN-HVF---KK---------VI--KVKNPPGCISDEAIESIK-----YLSTINDRNIALLVDGEED 110
YP_003458341.1 TV---IENN-IIPK---LSIFDLKTKRNIP--VEIN-HIF---KK---------II--KVKNPAGCISDEAIESIK----Y-LSTINDKDIALLVDGEED 110
YP_008075056.1 NL---ISHN-IYPD---VGIIDNRIQRKDH--DSII-NYT---DN---------IL--TAVNPAGTITDDLWETIE--FAIKQSIENNTRYIIDVDGEED 119
YP_001272768.1 NL---TKYE-LYPD---IGIIDNLIQRKNH--NHEV-IKA---EN---------IL--KAKNPAGTITDDLWETIG--QAMKLS-GDGNCHVIEVDGEED 122
YP_003424221.1 NL---LDND-LYPN---LGIIDNRIQRKDH--NHDI-IRT---EN---------IL--KADNPAGTITEDLWETIE--LAISSILEDEDTRIIEVKGEED 119
YP_004004002.1 NL---VDRG-IYPD---VAIIDRKIQRKKS--YYRI-EYK---SK---------IL--NVSNPPGTITESLMNAIK--KAL----KSKEKTIINVSGEED 115
NP_275409.1    NA---LEHG-LKPD---VSIIDNRIQRRDS--DHEF-RDT---AT---------IL--RSRNPPGTVTESLWKSIE--EAIEGATERGERFMIVVDGEED 116
YP_003849624.1 NA---LEHG-LKPD---VSIIDNRIQRMDS--DHEF-NDT---AT---------II--RSTNPPGTITEDLWNSIK--KAIDGA-RAGERFMIVVDGEED 115
YP_447663.1    NL---IKLN-IQPQ---ICIVDNKIEREPV--EHKL-THT---DN---------LV--HVNNPAGCITDELVKICI--DSINTA-TSNNPVIIEVKGEED 122
YP_004289823.1 KM---LDEG-IIPD---VGIVDNLIEREPS--DYEI-CYD---NV---------TL--KTKNPPGTITDQMWKTIK--EGFSLVEKAGYNVLIVVDGEED 122
YP_004520574.1 NM---LDAD-IIPD---MGIVDNKIERKPS--ENEI-YYD---NI---------TL--KAKNPAGTITDELQNTIE--KSFNLIENEGYKVLIIVEGEED 123
YP_008916228.1 NL---QKAD-LIPH---LGIIDNVVERKPA--AYDV-VYD---NV---------TL--NVKNPPGVITDELQETIK--RAFQLV-KSGFRVLILVDGEED 121
YP_003482939.1 TL---LNRG-IKPK---IAIVDYKTKRGEK--IYEDVKKF---GK--------KII--KVKNPRSQITPELWNAVR--EA-----MQYDSVKIEVDGEED 122
YP_007246537.1 TL---LKMG-IKPK---MAVVDYKTKRGEM--VFED-VRS---FG-------ERVF--RVENPAGTITPGLWDAVK--KAL----ISEKNVKIVVDGEED 123
YP_004341579.1 SL---LSMG-RKPV---IIVFDGRTER-RK--FEQL-EEL---ER---MTAEYERI--EVENPPSTITYELVVALK--RAVEVA-AEGVRTKVFVKGEED 115
YP_843741.1    YL---LKAS-FIPD---LMIVDHKTKRSPV--EDSI-KRRHLEGS-------YRVV--NVENPAGTLTEELLDAVR--ESL----NGCTPTEIIVDGEED 127
YP_004385292.1 YL---LKSS-IVPD---LAVVDNKTKR-AQ--LPDH-ILQ---SL---EHNSYKRV--EVKNPPATLTEELIDQIK--DSL----AGKERVKIVVEGEED 115
YP_005920892.1 CL---LSSSGRKPD---ICIVDHKTKR-MP--VPDH-VQQ---GI--GDRDEYDTI--EVYNPAATLTQELVDVIR--ERL----AGNGRVKIVVDGEED 129
YP_685355.1    YL---IKAG-VVPD---MCLVDDQTMR-LP--VDHE-VRK---GT---SHHTFKEL--RVSNPAGVVTQALMDLIK--DNM----SSTEPVRIFVDGEED 126
YP_003357728.1 HL---LKAG-IVPD---MCIVDDMTMR-LP--VAHE-VRK---GT---AHESFVDI--VVKNPAGVITQELIDAIK--NNM----ASPRPVRIFVDGEED 126
YP_005379469.1 FL---LKSG-RVPD---MCIVDDLTMR-LP--VDIE-VKK---GT---AHESFMDV--RVKNPAGVVTEELIGAIR--DNM----YSERPVRIYVEGEED 126
YP_303847.1    HL---LEVG-IIPD---ICIVDNRTKR-KP--VSSD-VSA---RN---MDKVYSEV--SVDNPAGIITDELIKTLC--EAF----ASEKPIRIFVRGEED 128
NP_618569.1    HL---LEAG-IIPD---ICIVDNRTKR-KP--VSRD-VSD---RN---RDKVYEEV--SVDNPAGIITDELIKTLC--EAF----DSEKLLRIFVRGEED 128
NP_632622.1    HL---LEAG-IIPD---ICIVDDRTKR-KP--VSSD-VSV---RN---RDKVYEEV--SVDNPAGIITDELIRTLC--EAF----ASEKLLRIFVRGEED 128
YP_007489295.1 HL---LEAG-IIPD---ICIVDDRTKR-KP--VSSD-VSV---RN---RDKVYEEV--SVDNPAGIITDELIRTLC--EAF----ASEKLLRIFVRGEED 128
YP_003726277.1 HL---IESG-IIPD---VYLIDNRTNRKPT--PDRV-DKN---HR-------FTQV--TVDNPSGTITEDLVNNIE--NAV----LSDKNVRIFVNGEED 125
YP_003541665.1 HL---LKSN-IIPD---ILVIDDKTHRQPA--CEQM-ITG---TK----QSGFKEL--VVNNPAGSITEELVDAFA--EAL----DSNRRVRIFVQGEED 128
YP_004615575.1 HL---LDSG-IIPD---ICIVDDRTKREPA--SEKV-VSG---TR----HTGFKEI--TVTNPAGIITEDLINVIN--YAF----ESDQNIRIFVYGEED 128
YP_566892.1    HL---LDSN-IIPD---ILIVDDRTKRAPA--SSQV-VFG---TK----HKGFAEI--TVDNPPGVITEDLINVIS--DAI----VSDKNVRIFVQGEED 122
YP_006922550.1 HL---LSSD-IIPD---ILIVDDRTKRGPA--SDRV-VVG---TK----HKGFREI--SVDNPAGVITGDLIDAVG--DAI----ASNCRVRIFVRGEED 122
YP_007312351.1 HL---LNSN-IIPD---ILVVDDRTKRGPA--SAHV-VVG---TK----HTGFSEI--FVDNPPGVITEDLINVVH--DAI----ISKDHVRIFVRGEED 128
YP_657114.1    HL---QTVD-YTPA---VAVIDGQTKR-ES--VDET-VKA---AL----SKHNKRI--DVENQPGTISIALLEALQ--TAV----ETPESVMIVVDGEED 121
YP_005839125.1 HL---QTVD-YTPA---VAVIDGQTKRESV--DETVKPALSKHNK---------RI--DVENQPGTISIALLEALQ--TAV----ETPESVMIVVDGEED 121
```

Fig. S6

```
YP_008055518.1 HF---EQAD-HQPA---VAFVDGRTKRETA--PDDVRAELDERPN---------RI--TVGNPAGVITADLLGAIA--DAL----DTDGPTTIVVDGEED 137
YP_001689848.1 HL---VDAG-QTPA---VAVVDGRTERR-DA--VRPA-VRD---AI------PEPDL--TVASEPGTVSVSLVRALV--DAI----ADADATVVSVDGEED 121
YP_003735962.1 HF---ERIG-RTPD---VAVVDGLTERETV--DPEV-AAALEGSD-------ARRI--AATNPAGALSESICRALR--EAI----ESDEPVAIDVAGEED 124
YP_002567039.1 HL---REAG-RVPD---VALIDGKTERR-ET--VDAE-IGS---AL---AAADDRRL--SVENPAASLSAELLEALS--EAL----SDADPVIIEVTGEED 128
YP_007285258.1 HV---LEAG-RTPD---VAIVDGITKRR-EA--VDAV-IRE---TV-----TAGETV--DVENPQGALTDALIRALV--AAI----DDPDPVTILVDGEED 133
YP_004808741.1 HL---RMAG-HEPH---VAVVDGRTKRR-ER--VTDE-IRD---AV---DAAGGRRV--EIRNEPAELSRDLLVALR--DAI----DHEESSLLLVEGEED 118
YP_004035542.1 HL---RRAG-RTPD---VAVIDGKTKRR-EA--VDEE-IRR---AL------SDPETRRDVNNPAGEITEALADTIR--DAL----DADDPVTVVVAGEED 117
YP_003535927.1 HL---RRAG-RPPD---VAVVDGKTKRR-EA--VGEE-IRR---AV-----ETGRLV--EVENEPGTVSVALVEALV--AAL----DDPDPTTLLVEGEED 121
YP_006349668.1 HL---RRAG-RPPD---VAIIDGKTKRR-EA--VDEE-IRR---AI-----ETGRLV--EVTNEPGTLSFDLVSALV--EAL----DAEEPTTLLVTGEED 121
YP_007486518.1 HL---LEAG-RRPD---VAVVDGKTKRR-ER--VERE-VLG---AI----EAFDDRI--DVANPQSTITDDLLEALA--SAL----EAPGSTVVVVDGEED 121
YP_331261.1    HL---LEAG-HRPA---AAIVDGKTERERV--ERKV-LSA---IE-----EFDERI--DVANPQSTITDDLLEALS--TAL----DRPDPTVVVVDGEED 121
YP_003479628.1 HL---LQAG-RVPD---VAFVDERTKRR-DA--VDEE-IRD---TV-----ATEPTH--EAINPPAELSGDVIRALR--EGL----DDEEPATVLVDGEED 137
YP_007309925.1 HI---VRAG-RDPD---VALVDERTKRR-QL--VEAE-IRE---TV-------TERVNVEARNPPAELTSDVLETLR--ESL----EREEPTTVLVDGEED 130
YP_003404991.1 HF---LRAG-RPPD---VALVDERTKRR-SA--VDEE-IRE---TV-----TEGTTV--EVVNPPAEISQAVVEALL--EGL----ASDEPTTILVEGEED 152
YP_006541826.1 HL---LQAG-HRPA---VALVDGRTKRR-TA--VDEE-IRD---AV-----TEGASI--EVRNPPAELGAPVVRALR--RAL----AADDPTTILVDGEED 131
YP_007279381.1 HL---LQAG-RRPD---VALVDGRTKRRTE---VDAE-IRE---AV-----TEGASI--EVRNPPAELGVPVVRALR--RAL----STGDPTTILVDGEED 131
YP_004597979.1 HL---LEAG-RAPD---VALVDERTERR-EA--VNED-VRE---TI-----TAETHL--EAVNPPAEISADVIEALL--EAL----SRDEPTTILVAGEED 146
YP_007177942.1 HL---LEAG-HEPD---LALVDERTERR-EA--VDEE-IRE---TV-----TARTNL--EAVNPPAEITEDVVWALC--EGL----ERLEPTTIVVDGEED 137
YP_003131105.1 HL---LEAG-RPPD---VSLIDERTERR-SA--VDAG-IRE---RIVEDEGWFEYRV--QVDNPAGTVTAALLGALV--EA--LD-RDEPSTIVVVDGEED 122
YP_008377469.1 HL---LEAG-RPPD---VSLIDERTERR-CA--VEAG-VRDRIVGTDADEEWFEYRV--QVDNPAGTVTADLLAALA--EA--LD-REDPSTIVVVDGEED 145
YP_003176564.1 HL---LEAG-YTPA---VALVDDRTKRR-AA--VDPE-VRD---AV----GGFDHCW--TVDNPAGTITGPLIGALS--RGL----GEDGTTLIRVDGEED 121
YP_004794731.1 HL---IEAG-RVPD---LALVDERTKRR-SA--VDAD-VSA---AI----DGFDRKR--DVSNPAATLTAELLAALR--DGI----DSGETTLLDVDGEED 121
YP_008874633.1 HL---IEAG-RVPD---LALVDERTKRR-SA--VDAD-VSA---AI----DGFDRKR--DVSNPAATLTAELLAALR--DGI----DSGETTLLDVDGEED 121
YP_504276.1    NL---LRAG-RPPE---VAIVDGHTMRQPY--PGVN-IPE---YH---------QL--LVKNPAGGLTEDLIDATQ--IA-----ASRQGTVIQVEGEED 113
YP_001030096.1 NA---LSAG-LIPS---IGVIDGFTKRSPY--LKMP-EIQ---GH---------IL--HVTNPAGTITDELIAALR--LA-----MDEIPCVVMVNGEED 113
YP_003893312.1 NL---VRND-VVPE---IAVIDGITMREPC--SRTPRLRS---SR----------F--EVENPPGMITGELIDTLL--LA-----LESRPALVHVNGEED 114
YP_001403408.1 SL---QQNG-ITPA---IAVVDGQTMRSPC--IKMP-EIA---GP---------CI--HVKNPPGTITDELVSALT--HA-----VDHTPVTILVDGEED 113
YP_007250359.1 TL---RKAG-ILPA---VGIIDGYTMRSPC--NRAPEVPG----E---------SL--RVKNPAGTLTDELVAAIR--YA-----VEHPPLTILVDGEED 113
YP_002465695.1 NI---LAAG-ILPA---IAIIDGYTRRLPC--PPAL-LHQ---AR---------QI--LVKNPAGAITDELIAAIG--DA-----AANPPALIVVEGEED 113
YP_001048149.1 NL---LAAG-VVPD---IAIIDGYTMRTPC--NRSP-LLR---AR---------RL--TVKNPPGTITGELTDAID--EA-----VRGQPAVIFVDGEED 113
YP_006545055.1 NL---LSAG-IVPD---IAIIDGYTMRTPC--TRSP-LLQ---AR---------RI--TAKNPAGTITDELVDAID--EV-----IRDPPGVIFVDGEED 113
YP_002582924.1 NV---IRLG-VYPS---VAIYDHRTKRKAY--NPSV-GSE---AV---------FL--TVKNPPGTITKALLNAVR--KGVEIA-GRGRRVHIKVNGEED 128
YP_002958867.1 NV---IRLG-MHPS---VAIYDHRTKRRDY--NPSV-GSE---AV---------FL--TVRNPPGTITKALLNAVR--KGVEIA-GRGRSVHIKVNGEED 128
YP_002308299.1 NV---LRLG-IKPS---LAIYDHKTKRQEY--SPDI-ESD---AV---------IM--TVQNPPGTITKALLNAIK--KGFGLA-ERGRKVYIKVCGEED 125
YP_004762704.1 NV---LKLG-IRPS---IAIYDHRTKRHEY--NPDI-ETG---SV---------VM--TVQNPPGTITKALLNAIR--KGFGLA-ARGRRVYIKVYGEED 124
YP_006424759.1 NV---IKLG-ISPS---LAIYDHRTKRKAY--NPSI-DPG---AV---------VM--TVQNPAGTITKALLNAIR--KGFGLA-ERGRRVYIKVNGEED 124
YP_004072211.1 NV---LKLG-IKPS---VAIYDHKTERNEY--NPEI-EFG---AV---------IL--TANNPPATITKALLNAIR--KSIELA-KRGLNVYIKVHGEED 128
YP_002993448.1 NV---LKVG-IKPS---VAIYDYKTKRKEY--EPDI-EFG---AV---------VL--TTKNPPGTITKALLNAVK--KSFELA-KKGKRVYLKVNGEED 128
YP_008428988.1 NV---LRLG-IKPS---IAIYDHKTKRREY--EPDI-ELG---AV---------VL--TTKNPPGTITKALLNAIK--KSFELA-KRGRRVYIKVNGEED 128
YP_004624278.1 NV---LKLG-VKPS---IAIYDHRTERRDY--KPDI-EPD---AV---------VI--TVQNPPGTITKELLEAIR--KAYELV-GRGKRVYIIVGGEED 126
YP_006354082.1 NI---LKVG-LTPN---LAIYDHKTERREY--RPDI-KTN---AI---------ML--TVKNPPGMITLSLLKAIK--KAYLLV-NRGKNVHIIINGEED 124
YP_004423817.1 NV---LKVG-LNPV---LAIYDHKTERREY--KPKI-NVN---GV---------IM--TVKNPPGVITLPLLKVIK--KAYSLI-SSGKRVHIIINGEED 126
NP_577983.1    NS---LKVG-LRPR---LAIYDHKTERREY--KPEI-GDK---GV---------LL--TVKNPPGTITLHLLKTIK--KAYSLI-RRGTNVHIVVNGEED 125
YP_006491333.1 NS---LKVG-LRPR---LAIYDHKTERREY--KPEI-GDK---GV---------LL--TVKNPPGTITLHLLKTIK--KAYSLI-RRGTNVHIVVNGEED 125
NP_127361.1    NI---MKVG-LNPN---LAIYDHKTERREY--KPNIRSVE---GV---------LI--TVKNPPGTITLPLLKAIK--KAYSLL-SHGKRVHIVVDGEED 127
NP_143738.1    NI---MRVG-LNPN---LAIYDHKTERREY--KPRI-IIN---GV---------LL--TVKNPPGTITLPLLKSIK--KAYSLI-LNGKSVHIVVNGEED 127
Jpred 4 SS     HH---HH------E---EEEEE--E-------------------E---------EE--EEE------HHHHHHHHH--HHHHHH------EEEEEE----
TK1697         NV---LKIG-VKPI---IALYDLKTKRKEY--SPEI-EDT---AV---------FL--TVTNPPGTITKALLDTVR--KAFGLA-ERGRNVHILVSGEED
CONSENSUS 0.95 ---------------------D----R----------------------------------N--------------------------------V-GEED

YP_919986.1    LLSLVALLYCPLNDS-WVIYGNFR-GYLEVIPCTSFFRSVAENLLVKHFEKD------------------------------------------------ 175
YP_003816556.1 MLTLAALSCAPANSL--IIYGIPN-RGAAVVVTNTIISHEVQTRL-LRLVPSSIMVQA------------------------------------------ 156
YP_007174639.1 LLALPTLSCAQDNSL--VIYGIPN-KGAALVNVNNYIRRELQNKI-LVLKPGIIETK------------------------------------------- 177
NP_147085.1    LIALAALDCGIDWT---VVYGLPGVGGVVVHRCLRKPGLENSSVL--AFKPGTGVHHQSSP--------------------------------------- 185
YP_008603597.1 LAALAALDCGSGWT---IVYGLPGVGGVVVHNCLRKPGVGSSGII--AFKPGVVRLKA------------------------------------------ 182
YP_006363113.1 LLTTSLAWVLSQGT---VAYGQPG-VGVVSVEVDKVRLAKFIKVL-KPAIIHITPGRA------------------------------------------ 158
YP_001040844.1 LVTLAYALENEETS---IAYGQPD-IGVVII---KSNRLKALRIL-KTFKPDIVVYNKV----------------------------------------- 160
YP_003669616.1 LVTLAYALENNETS---IAYGQPD-IGVVII---KNNRFKALRIL-KTFKPDIVVYNKV----------------------------------------- 160
YP_003650585.1 LLVFPLALPSTGVSS--IIYGQPN-VGVVEL---DARGIRILKLL-KTFKPVNGFI-------------------------------------------- 161
YP_004177111.1 LLVIPFTL--TWGS---VIYGQPD-TGVVK-TASRERALKILKGL-KPHMAIINLKGEQRNG-------------------------------------- 163
YP_002429036.1 LLVIPFTLVRGFK----IIYGQPD-VGVV---ISSPSRERVLKIL-KGLKPDIVIMNL------------------------------------------ 157
YP_006402879.1 LLVIPFTLVRGFK----IIYGQPD-VGVV---ISSPSRERVLKIL-KGLKPDIVIMNL------------------------------------------ 157
YP_004780378.1 LTAIPAIIEAPDGAV--IAYGVPW-SGLVVVEA-DWARAVALHIV-NASMLVKCR--------------------------------------------- 175
YP_005841883.1 LLSLYVPLLIPMNSSGRVIYGQPG-MGAVVFDVNEKTKREISNIL-QDFYIEFSI--------------------------------------------- 174
YP_930015.1    MLSLAFIKLAPPHSV--VVYGHYM-GALIAIPV-DWYRDAICKLF-EYLEKC------------------------------------------------ 168
YP_001794902.1 MLALAFIKMAPPRSL--VVYGHY--KGALIAVMVDWYRDAIDRLL-QYLEKC------------------------------------------------ 168
YP_001056647.1 MLSLAFIRLAPPRSI--VAYGHYM-GALIAIPV-DWYRRDLLKLF-DFLEKC------------------------------------------------ 168
YP_001153914.1 MLSLAFIKLAPPRSV--VAYGHYL-GALVAIPV-DWYRDSILRLF-NYLEQCQQKA-------------------------------------------- 172
YP_005259057.1 MLSLAFIKLAPPRSV--VAYGHYL-GALVAIPV-DWYRDSILRLF-NYLEQCQQKA-------------------------------------------- 172
NP_560628.1    MLSLAFIKLAPPKSI--VAYGHYL-GALIALPV-DWYKEYVLKLF-DYLEKC------------------------------------------------ 140
YP_005085004.1 MLSLAFIKLAPPRSI--VAYGHYM-GALIAVPV-DWYRSYILKLF-DYLEKC------------------------------------------------ 168
YP_004337001.1 MLSLAFIALAPDDSA--VVYGHYK-GALIVIPVGKY--KEIRELL-KYMEPC------------------------------------------------ 166
YP_004892291.1 MLSLAFIALASDDSA--VVYGHYK-GALVIIPVGAY--REIKSLL-KFLKEC------------------------------------------------ 169
YP_008796927.1 LLALPAIHALPDGGV--VVYGQPR-QGYVVVKASVKVKKLVEEII-SMATV------------------------------------------------- 168
YP_003860570.1 LLTLATIILIDHG-I--VVYGVPH-EGIAIIPI-DKAKVDAINIF-SQFIRLNSLGYKDLNYV------------------------------------- 201
YP_008071435.1 LAALVCAAAAPTGSC--LMYGVPG-VGMALLKVDAASSDRAKSLL-YSMEELN----------------------------------------------- 160
YP_007687666.1 LALLPCVLHAPEGAV--VVYGWPG-RGMKAVATDGSSRKWAEEML-GRMEELS----------------------------------------------- 168
YP_007713106.1 LALLPCVLYAPEGAV--VIYGWPG-RGMKAVDTDGLSKAYIEKLL-ESMEELI----------------------------------------------- 171
YP_001541769.1 LLAIPAVLEAPGNTG--ILYGLYT-GYLVLIPAVNEYKILMLKLL-TLLDRDECETLRNCNSVNNYGWKNS----------------------------- 200
YP_003902647.1 LLAIPAVLLAPRGSL--VLYGLYT-GYLIAIPITDEYKLAMLKLF-SMMKPANA---------------------------------------------- 175
YP_004244378.1 LLAIPAVLLAPKGSL--VLYGLYT-GYLIAIPVVDEYKLAMFKLF-LMMRPGNA---------------------------------------------- 174
YP_001737791.1 LLGFPAIILPPEGWV--MVYGQPG-VGMVSVNIDRKAREEAMNLLQEAFLPI------------------------------------------------ 167
YP_023646.1    LAVIPIIFYGDINTL--VVYGVPD-TGMACIFINTEIKSMVTDIL-RDLNGKT----------------------------------------------- 165
YP_008142358.1 LAVIPIIFYADKNTV--IVYGVPD-VGMAYIKVNDEIKERIKEMI-MEMYRNEQ---------------------------------------------- 168
NP_111003.1    LAVLPIIYYSKNNTV--IAYGIPD-VGMAALTVTDDLRDHVTEVI-AKMAIE------------------------------------------------ 166
NP_394550.1    LAVLPIIYYADLDTV--IAYGIPD-VGMAILTVDFDLKCHVCEVL-SEMAIA------------------------------------------------ 156
NP_341954.1    LLVIPVTLSANHGDI--VIYGQPN-AGAVVIIVNEMIRWRVRDIL-EKAIVKEC---------------------------------------------- 179
YP_005643228.1 LLVIPVTLSANHGDI--VIYGQPN-AGAVVIIVNEMIRWRVRDIL-EKAIVKEC---------------------------------------------- 179
YP_002915007.1 LLVIPVILSANNGDI--VIYGQPN-AGAVVIIVNEMIKWRVRDIL-EKAVVEEC---------------------------------------------- 179
YP_002829735.1 LLVIPVILSANNGDI--VIYGQPN-AGAVVIIVNEMIKWRVRDIL-EKAVVEEC---------------------------------------------- 179
YP_002843724.1 LLVIPVILSANNGDI--VIYGQPN-AGAVVIIVNEMIKWRVRDIL-EKAVVEEC---------------------------------------------- 179
YP_005648862.1 LLVIPVILSASNGDI--VIYGQPN-AGAVVIIVNEMIKWRVRDIL-EKAVVEEC---------------------------------------------- 179
YP_005646225.1 LLVIPVILSANNGDI--VIYGQPN-AGAVVIIVNEMIKWRVRDIL-EKAVVEEC---------------------------------------------- 179
YP_007866027.1 LLVIPVILSANNGDI--VIYGQPN-AGAVVIIVNEMIKWRVRDIL-EKAVVEEC---------------------------------------------- 179
```

Fig. S6

```
YP_002832457.1 LLVIPVTLSADNGDI--VIYGQPN-AGAVVIIVNEMIRWRVRDIL-EKAVVKEC---------------------------------------------- 178
YP_002840150.1 LLVIPVTLSADNGDI--VIYGQPN-AGAVVIIVNEMIRWRVRDIL-EKAVVKEC---------------------------------------------- 178
YP_003419971.1 LLVIPVTLSADNGDI--VIYGQPN-AGAVVIIVNEMIRWRVRDIL-EKAVVKEC---------------------------------------------- 178
YP_002837983.1 LLVIPVTLSADNGDI--VIYGQPN-AGAVVIIVNEMIRWRVRDIL-EKAVVKEC---------------------------------------------- 178
YP_004457895.1 LLVIPATLYGRDGDL--VIYGQPN-AGAVALEVCEATKWRVNDIF-SKFNVKKC---------------------------------------------- 174
YP_001192301.1 MMVIPIILYGKNGDL--VVYGQPN-AGAVCLENWEGSRWRVMDIF-SKFTAELC---------------------------------------------- 173
YP_004408663.1 MMVIPVIMYGKDGDL--IIYGQPN-AGAVCLENWSGSRWRVRDIL-SKFTVEPC---------------------------------------------- 111
NP_376249.1    LLVIPVVIFGRNNDI--IVYGQPN-AGAVVIINNHFVKIRVKQIL-EKFYVKKC---------------------------------------------- 162
YP_007433930.1 LLVIPVVIYGNFGDI--IIYGQPN-AGVVVTLNNDFLKRRVLEIF-KKFQVTSC---------------------------------------------- 164
YP_007436201.1 LLVIPVVIYGNFGDI--IIYGQPN-AGVVVTLNNDFLKRRVLEIF-KKFQVTSC---------------------------------------------- 164
YP_255496.1    LLVIPVVIYGNFGDI--IIYGQPN-AGVVVTLNNDFLKRRVLEIF-KKFQVTSC---------------------------------------------- 164
YP_008946885.1 LLVIPVIIYGNFGDM--IIYGQPN-AGVVLMLNNDFIKRRVLEIF-KKFQITSC---------------------------------------------- 178
YP_001012648.1 LLALPALLYGDVGSV--VLYGLPG-KGVVAAAVNREAKLLAMRVL-EFFEPC------------------------------------------------ 125
YP_006862428.1 MLALPLFTMAPEGSA--VLYGQPL-EGLVIVKITSAKQKQAKDLM-DRIGID------------------------------------------------ 166
YP_875902.1    LLVIPACIYAPDGAV--IMYGQPG-RGLVAIHVDAGIRYKAKGLL-DSVS-------------------------------------------------- 151
YP_006776123.1 LLVLPVCVHAPENAV--VLYGQPH-EGLVIVKITPEIRNKVQTLL-DLME-------------------------------------------------- 154
YP_001582567.1 LLVLPVCIHAPENSV--VMYGQPN-EGLVIVKITSEIRNKVQSLL-DLME-------------------------------------------------- 154
YP_006774212.1 LLVLPVCIHAPENSV--VLYGQPN-EGLVVVKINSEIRNKVQSLL-DLME-------------------------------------------------- 164
ZP_08257196.1  LLVLPVCVHAPENSL--VMYGQPN-EGLVLVNITPEIRNKTQGLL-DLME-------------------------------------------------- 165
ZP_08668423.1  LLVIPVCIHAPENSV--VLYGQPN-EGLVTVKVTPEIRNKTQRLL-DSME-------------------------------------------------- 151
YP_003400341.1 LAVMPLVALLPNGAV--ILYGQPN-EGVVRVDVNDERKIIILSML-ERMEKHGKTLEKIRRWSNGGSR-------------------------------- 190
YP_003436628.1 LATLVLAALMPSGSI--ILYGIPS-VGIAAYEVGE--KVLILNLL-QKFEAEENDRVMKMIEEVLEWN-------------------------------- 190
NP_069944.1    LAVVPLVKLLPLGAL--ILYGQPG-EGVVALKVDEEKKVLILNLL-NKMEIIGECDELKYLLGGD----------------------------------- 186
YP_007906114.1 LAVLPLVYMLPLNSL--LLYGQPR-EGIVAVVISEEKKNIIPDIL-QRMEKLEDGDLVMDLVMGEGSG-------------------------------- 195
YP_001434673.1 LLAIPALLSSENGWA--LLYGQPK-AGVVLVEINKYTKLHFLEII-KMFDGDVEEFLREFDYDPNQPLLGELDERLYDLLFPEL---------------- 184
NP_614736.1    LLAIPAILIAPENSI--VCYGLPG-EGMVAARVTQHLKDSVLRLL-TRFRGYDEWKSRSWISGITPCCTAKR---------------------------- 191
YP_003706636.1 LLTIPVIIYFPIGCY--IFYGQPD-EGVVVVKITEELKHYANEII-DKFEKKNI---------------------------------------------- 172
YP_001323935.1 LLTLPVIKYFPEDTS--VIYGQPD-EGMVVLKITKELKQKIEKLL-KDMEER------------------------------------------------ 158
YP_001330653.1 LLTIPVIKYFPEDTS--VIYGQPD-EGMVLLKITDELKQKIEKLL-KDMEER------------------------------------------------ 158
YP_001097707.1 LLTIPVIKYFPEDTS--VIYGQPD-EGMVLLKITDELKQKIEKLL-KDMEER------------------------------------------------ 158
YP_004742083.1 LLTIPVIKYFPEDTS--VIYGQPD-EGMVLLKITDELKQKIEKLL-KEMEER------------------------------------------------ 158
NP_987562.1    LLTIPVIKYFPEDTS--VIYGQPD-EGMVLLKITDELKQKIEKLL-KEMEER------------------------------------------------ 158
YP_001548519.1 LLTIPVIKYFPEDTS--VIYGQPD-EGMVLLKITDELKQKIEKLL-NDMEER------------------------------------------------ 158
YP_001324483.1 LLTMMVIRYFPKDTI--VLYGQPD-EGVVLLTINQDLKHKINNIL-SMMDKI------------------------------------------------ 161
YP_004484635.1 LLVIPVIKYFPENTI--VLYGQPN-EGIVFLKITQELKNQIDEIL-KMMERR------------------------------------------------ 158
YP_004575995.1 LLTIPVIKYFPEDTI--VLYGQPN-EGVVLLKINKDLKNRIDEIL-NMMDKR------------------------------------------------ 158
YP_003616685.1 LLALLVIKYFPKNIY--VAYGLPD-KGVILLKIDDELKKKIDEIL-KKFEKVKMMNIKIVSERYNPLAHRKEIRFIVDHEGATPTFKDVKLKLAAMLNVN 206
YP_003247808.1 LLTLPVIKYYPEGHY--VLYGQPN-EGIVVLKIDKKLKQEIDNIL-KKFKKIDERDKDGNKNIVRQIQPTIEKDRV------------------------ 182
YP_003128192.1 LLALIVIKYFPIGTY--VIYGQPN-EGIVVLKIDEKLKQEVEETL-KQFKKL------------------------------------------------ 158
NP_247369.1    LLALIVIKYFPIGTY--VLYGQPD-EGIVVLKINKKLKQEIEEIL-KQFKKI------------------------------------------------ 158
YP_003458341.1 LLALIVIKYFPIGTY--VLYGQPD-EGIVVLKIDEKLKQEINEIL-KQFKKI------------------------------------------------ 158
YP_008075056.1 LAVLPIIALAPEYTT--VLYGQPN-EGLVLLNAGD-LKEKGEKFI-SDFNEI------------------------------------------------ 166
YP_001272768.1 LAVLPCILMASDETT--ILYGQPN-EGLVLLKVCD-AKDYAQNLI-DTFIKE------------------------------------------------ 169
YP_003424221.1 LAVLPCLLIAPEDAV--ILYGQPN-EGLVFVNVF-EAKDKATRLM-TFFNKE------------------------------------------------ 166
YP_004004002.1 LAVLPAILFSSKAL---ILYGQPN-EGVVVVDA-DKSKNKAKKII-KEFKEVKEHGNKYN---------------------------------------- 169
NP_275409.1    LAVLPSILLAPPDTV--ILYGQPD-EGVVLVRA-SETSSKAEELM-KKFEEA------------------------------------------------ 163
YP_003849624.1 LAVLPSILMAPADTV--ILYGQPN-EGVVLVRAI-ETRKRAEELI-KNFEEA------------------------------------------------ 162
YP_447663.1    LAVLPCILNAPKDTF--ILYGQPK-EGVVLVCVNEAF-NKAKHFY-KQLNKE------------------------------------------------ 169
YP_004289823.1 LSAIPCMVMAPEGSI--IFYGQPG-EGVVLCEV-DKTKEKAQDLI-KKLEEA------------------------------------------------ 169
YP_004520574.1 LAVIPCAIMAPSGSI--IFYGQPG-EGVVLCEV-DKVKKIAENLI-KKLEEA------------------------------------------------ 170
YP_008916228.1 LAAIPSILMAPPGSL--VLYGQPG-EGLVVCEV-DKVIDKVKQMK-NKLEER------------------------------------------------ 168
YP_003482939.1 LAVIPVVFFANLGAN--VIYGMPN-TGLVWLKVSDEDKRKVMEII-KEMEV------------------------------------------------- 169
YP_007246537.1 LAVIPVVFFAPLGAN--VIYGMPN-TGLVLLKITPEERDKVKDLI-KKMEV------------------------------------------------- 170
YP_004341579.1 LALMPLVCLLPSGS---VVYGQPG-EGVVEVNVTPEKKLLILQLM-EQMERLKRDGKNGEDVISLCRGWSNGDLR------------------------- 185
YP_843741.1    LAALPAILYAPLGSA--VVYGQPS-VGSVLVMVTPEKKREIEGIL-RRMTVKEKV--------------------------------------------- 178
YP_004385292.1 LATLPAILYAPPGSV--VIYGQPD-EGSVLVKATPEMKMYIDELM-KKMIVEE----------------------------------------------- 164
YP_005920892.1 LATLPAILYAPLGST--VVYGQPN-EGSVAVVVTPERKEYAKSIM-DKMIVED----------------------------------------------- 178
YP_685355.1    LAVIPACLYAPIGSA--VIYGQPN-EGVVVVRVTEEKRKETKALL-DKMERVDE---------------------------------------------- 176
YP_003357728.1 LAVIPACEYAPAGSL--VLYGQPK-EGVVAVEVTPEKKRETLSLM-EQMVRSNNSVTE------------------------------------------ 180
YP_005379469.1 LAVIPACVYAPVGSV--ILYGQPS-EGVVAVRVTEDKKRETCSLM-EQMIKE------------------------------------------------ 174
YP_303847.1    LATLPVILLAPLDAV--VLYGQPD-EGVVFVKVTEEKKGEIRTLF-EKLISKNQNYELDKLRRILDGHKNS----------------------------- 195
NP_618569.1    LATLPVILMAPLGSV--VLYGQPD-EGVVFVRVTEEKKEEIRVLF-EKLISKNQNTELDKIRRILDGHKDP----------------------------- 195
NP_632622.1    LATLPVILLAPPGSV--VLYGQPD-EGVVFVEVTEKKKEEIRALF-EKLISKNQNNELDKIRRILDGHKDN----------------------------- 195
YP_007489295.1 LATLPVILLAPPGSV--VLYGQPD-EGVVFVEVTEKKKEEIRALF-EKLISKNQNNELDKIRRILDGHKDN----------------------------- 195
YP_003726277.1 LAALPAILLAPINSI--VLYGQPG-EGVVLVEITESKKEEIRDLMNNIIEFQNTNNSLNKLRRKLNGNKNK---------------------------- 193
YP_003541665.1 LASLPAIMMAPAGSV--VLYGQPG-EGMMFVRITASKKEEMTELIDKIIDKQHCKEQLYTMWRKLYGYQHN---------------------------- 196
YP_004615575.1 LAALPSILMAPICSV--VLYGQPG-EGIVMVKITRPKKIEIQKLIDSIIEQPENKDKLHSNIWRKLNGH------------------------------ 194
YP_566892.1    LAALPAILMAPLNSV--VLYGQPD-EGVMLVRVTESLKAELKDLFDKILEKQDHKEQLLHNVRRKLNGY------------------------------ 188
YP_006922550.1 LAAVPAMLMAPEGSA--VLYGQPD-QGVVLVQITESKKEEIRDLL-TKIIEAQGHNEDTETIWRKLDGYKDHKG-------------------------- 192
YP_007312351.1 LAALPAILLAPEGSV--VLYGQPD-EGVVLVKITKSKKEEILDLL-DKMIHGQEERNSLIDIRRKFNGY------------------------------- 193
YP_657114.1    LATLPAVLVARPGGT--VVYGQPD-QGMVRIAVTPETKITMSRLL-KRMDGDAAAAFDRLGVDRSGDKK------------------------------- 186
YP_005839125.1 LATLPAVLVARPGGT--VVYGQPN-QGMVRIAVTPETKTTMSRLL-KRMDGDAAAAFDRLGVDRSGNKK------------------------------- 186
YP_008055518.1 LATVPVVLVAPLGGT--VVYGQPD-EGMVAVDVTEDHRAQFRDLL-STFDGDVERALQLLGV-------------------------------------- 195
YP_001689848.1 LAVVPAVLAAPADAT--VVYGQPG-EGMVRVPVTDAGRAEMRERA-DRLETTAAFWRLVD---------------------------------------- 177
YP_003735962.1 LVTVPAIALAPDGAS--VVYGQPG-EGMVHVRIDGETRNRARDLV-GRMDGDHERLWELLEG-------------------------------------- 182
YP_002567039.1 LAALPAILAAPDGAS--VVYGQPG-EGMVRVAVTPESRTEARELF-EALDGDVEAAYKALGRVPDGDR-------------------------------- 192
YP_007285258.1 LATLPAIVAAPEGAS--VVYGQPD-KGMVHAVVTDERRAAIRSLL-TRFDGDPEALFSILDR-------------------------------------- 191
YP_004808741.1 LATLPAVLAAPLGAT--VVYGQPD-EGMVAIPVTEETRAAMRELL-AKFDGDADAAMATLGV-------------------------------------- 176
YP_004035542.1 LATLPAVVAAPTGAS--VVYGQPD-EGMVLIDVTDDSKAEMRSLL-RRMDGDAESLLSLLES-------------------------------------- 175
YP_003535927.1 LATLPAVLAAPVGST--VVYGQPD-EGMVRVAVTDAAKSEMRDLL-ARFDGDFDAVVAPIDA-------------------------------------- 179
YP_006349668.1 LATLPAVLAAPLGST--VVYGQPD-EGMVRVTVTEAAKTEMRDLL-AQFDGDIETVLSPLDT-------------------------------------- 179
YP_007486518.1 LASLPAVVGAPDDAS--VVYGQPD-EGMVLVPVEEESRVRCRELI-EGMQSDYDRIAAILS--------------------------------------- 178
YP_331261.1    LASLPAVVAAPVGAS--VVYGQPG-EGMVLVHVTDETQAECRDII-ERMQSDYSQIESILS--------------------------------------- 178
YP_003479628.1 LVAVPAIAAAPEGAS--VVYGQPD-EGMVHVRVTSEHRTEMRELL-ERFDGDVERLWTLLESPVDDA--------------------------------- 200
YP_007309925.1 LVVLPAIMVAPDGAS--VVYGQPD-EGMVHVVVDDEVRREVRALL-ERFEGDQERFWELLEE-------------------------------------- 188
YP_003404991.1 LVALPAIVAAPEGAS--VVYGQPD-EGMVHVKVTEEHQQDMRALL-DRFEGDIDRFWELLEASGG----------------------------------- 213
YP_006541826.1 LVALPAIVAAPENAS--VVYGQPD-KGMVHVTVTDDHRAEMRDLL-ERFEGDTERFWELLEGSSDGE--------------------------------- 194
YP_007279381.1 LVALPAIVAAPEGAS--VVYGQPD-EGMVHVTVTDDHRAEMRDLL-ERFEGDTERFWELLEG-------------------------------------- 189
YP_004597979.1 LVALPAIVAAPEGAS--VVYGQPG-EGMVHVRVTDDHRERMRALL-ERFEGDTERLWSLLEERDE----------------------------------- 207
YP_007177942.1 LVALPAIVAAPDGAS--VVYGQPG-EGMVHVRVTEETRTRGRELL-ERFEGDIERFFQLLE--------------------------------------- 194
YP_003131105.1 LATLPAMVVAPPGAS--VVYGQPG-EGMVHVAVDTETSERARSIL-SRMDGDHARAWKLLGV-------------------------------------- 180
YP_008377469.1 LATLPAIIVAPPGAS--VVYGQPG-EGMVHVAADTAASERARSIL-SRMDGDQDRAFELLGIS------------------------------------- 204
YP_003176564.1 LAALPAVLAVPEGAS--VVYGQPD-EGMVLVTADAAARERCRDLI-GRMDGPADELLAALS--------------------------------------- 178
YP_004794731.1 LATLPAVLAAPAGAS--VVYGQPD-EGMVLADCDAEARDRVRSLL-ERMDGDAERAIALVSE-------------------------------------- 179
YP_008874633.1 LATLPAVLAAPAGAS--VVYGQPD-EGMVLADCDAEARDRVRSLL-ERMDGDAERAIALVSE-------------------------------------- 179
```

Fig. S6

```
YP_504276.1      LAVVPLAMHAPLGTV--ILYGQPG-EGVVLLSITPAMKKRAEELF-TCFEEVSTPTAREVFNI------------------------------------- 172
YP_001030096.1   LAVLPLTEILPDGAV--ILYGQPE-EGLVICEVNKQLRANAKKLL-TYFVSL------------------------------------------------ 161
YP_003893312.1   LAVIPLVKIVPDGSA--VFYGQPG-EGVVVKIVDEEARRLADELF-SLFEADNSCE-------------------------------------------- 166
YP_001403408.1   LAVIPLVIAAPLSSI--VIYGQPN-EGVVLRIVDDQAKTAARRLL-TQFTKTESPIPHN----------------------------------------- 168
YP_007250359.1   LAVIPMVIAAPLGSI--VLYGQPG-EGVVLRTVTPGAQDTARAFL-ERFIRSEE---------------------------------------------- 163
YP_002465695.1   LAVLPVILAVPDGGF--LLYGQPG-EGAVLCTVDQQAKERAREML-ALFEQV------------------------------------------------ 161
YP_001048149.1   LAVIPLVLAAPDGAA--VLYGQPA-EGVVLRLVDTAAKQEAASML-SIFVRE------------------------------------------------ 161
YP_006545055.1   LAVIPLVLAAPSGAA--VLYGQPG-EGVVLRIVDASAKREAETIL-KAFVRE------------------------------------------------ 161
YP_002582924.1   LAAIPAVLYAPLGSM--VLYGQPD-EGVVLIKVTPECKRRCARIL-AKMEVVRDGD-------------------------------------------- 180
YP_002958867.1   LAAIPAVLYAPLGSV--VLYGQPD-EGVVLIKVTPECKRRCASIL-AKMEVVRDGD-------------------------------------------- 180
YP_002308299.1   LAAIPAVLYAPEGSV--VLYGQPD-EGVVLIKVTPECKLKCGRIM-SKMEVVRDGD-------------------------------------------- 177
YP_004762704.1   LAAIPAVLYAPEGTL--VLYGQPD-EGVVLIKVTPECKLKCGKIM-SKMEVIHDGD-------------------------------------------- 176
YP_006424759.1   LAAIPAVLYAPPGSV--VLYGQPD-EGVVLIKVTPECKLKCGKIM-SKMEVVHDGD-------------------------------------------- 176
YP_004072211.1   LAAIPAVLYAPFNAL--VVYGQPK-EGVVLIKVTPECKRRCAEIL-RKMEVVYDGD-------------------------------------------- 180
YP_002993448.1   LAAIPAVIYAPEGAL--VIYGQPD-EGIVLIKVTSECKRRCAKIL-RKMEVVHDGD-------------------------------------------- 180
YP_008428988.1   LAAIPAVLYAPEGTL--VVYGQPD-EGIVLIKVTPECKRRCAQIL-RKMEVVYDGD-------------------------------------------- 180
YP_004624278.1   LATIPAVLYASPGTL--VVYGQPR-EGIVLIKVIPECKRRCAEIM-RRMEVVRDGD-------------------------------------------- 178
YP_006354082.1   LATIPAVLYAPYNSY--VIYGQPR-EGIVLIKVTHECKRRCAEIM-RKMEVVRNGD-------------------------------------------- 176
YP_004423817.1   LATIPAVLYAPYGAI--VIYGQPR-EGIVLIKVTEECKRRCAKIL-REMEVVKNGDQNYRD--------------------------------------- 183
NP_577983.1      LAAIPAVLYAPLGSF--VVYGQPR-EGIVLIKVTSECKRRCAEIM-RNMEVVRNGD-------------------------------------------- 177
YP_006491333.1   LAAIPAVLYAPLGSF--VVYGQPR-EGIVLIKVTSECKRRCAEIM-RNMEVVRNGD-------------------------------------------- 177
NP_127361.1      LATIPAVLYAPIGTT--VIYGQPK-KGIVLIKVTNECKRRCAKIM-RRMEVVRNGD-------------------------------------------- 179
NP_143738.1      LATIPAVLYAPLGAT--VIYGQPK-RGIVLIKVTNECKRRCAKIM-RRMEVVRDGD-------------------------------------------- 179
Jpred 4 SS       HHHHHHHHH-----E--EEEE-----EEEEEE--HHHHHHHHHHH-HHH---------------------------------------------------
TK1697           LAAIPAVLYAPLGTL--VLYGQPD-EGVVLIKVTPECKRRCAKIL-ASMEVVRDGD--------------------------------------------
CONSENSUS 0.95   L------------------YG-------------------------------------------------------------------------------
```

B

```
YP_004457895.1  -------------------------MEIRDKYKVDLCFYLP---KEVRKELSRPYGILFKS-----TEKLLKYVDN--------FERI--ITVGDVVTQEL--------LKNN-RRVFLSIVDGKTKRNIKLHMRFSN--YI---MVKNEPAIIRLSAMSYIK--AAL----NSSFSSVIYVDGEEDLLVIPATLYGRDGDLVIYGQPN-AGAVALEVCEATKWRVNDIF-SKFNVKKC--
TK1697          ---------------------------------MKMFFRLT---RELRDELKRPLGELVRGPIPEPYLKVRGELEK---------HPV--VTVGDVVTENV--------LKIG-VKPIIALYDLKTKRKEYSPEIEDTAVFL---TVTNPPGTITKALLDTVR--KAFGLA-ERGRNVHILVSGEEDLAAIPAVLYAPLGTLVLYGQPD-EGVVLIKVTPECKRRCAKIL-ASMEVVRDGD
Jpred 4 SS      -----------------------------------EEEE-----HHHHHHHH---EEE-----HHHHHHH------------EE--EEEEHHHHHH--------HH------EEEEEE--E------------EEE---EEE-----HHHHHHHHH--HHHHHH------EEEEEE----HHHHHHHHH-----EEEEE-----EEEEEE--HHHHHHHHHHH-HHH-------
CONSENSUS 0.95  ----------------------------------------------------------------------------------------------GD-----------------D----R------------------N-------------------------------V-GEEDL--------------YG----------------------------------

PDB: 3MEL       ----------------------------------------------------MSRVLLVAGGNP---SDWPTIEPAT--------YDYF--VGI-DRGCLHL--------LEAD-LPLQLAVGDFDSLSREEYHFVQET--TE--TLIQAPAEKDDTDTQLALQEALQRFP------QAEMTIIGATGGRIDHLLANLWLPFE
PDB: 3MEL SS    ------------------------------------------------------EEEEE-----------------------------E--EEE---HHHHH--------H--------EEEE--------HHHHHHHH-------EEE--------HHHHHHHHHHHH----------EEEEE------HHHHHHHH-----
PDB: 2F17       --------------------------SSGLVPRGSHMEHAFTPLEPLLPTGNLKYCLVVLNQPL---DARFRHLWKK--------ALLR--ACA-DGGANHLYDLTEGE--RES-FLPEFVSGDFDSIRPEVKEYYTKK--GCDLISTPDQDHTDFTKCLQVLQRKIEEKE----LQVDVIVTLGGLGGRFDQIMASVNTLFQ
PDB: 2F17 SS    --------------------------------------EEE-------------EEEEE-----------HHHHHHH----------EE--EEE---HHHHHHHH--------------EEEE------HHHHHHHHH-----EEEE-------HHHHHHHHHHHHHHH----------EEEEE------HHHHHHHHHHHHH
Catalytic aa                                                                                     *                          * **
```
